# Intrinsic and acquired drug resistance to LSD1 inhibitors in small cell lung cancer occurs through a TEAD4‐driven transcriptional state

**DOI:** 10.1002/1878-0261.13124

**Published:** 2021-11-09

**Authors:** Wen Yan, Chi‐Yeh Chung, Tao Xie, Mark Ozeck, Timothy C. Nichols, Jessica Frey, Akshata R. Udyavar, Shikhar Sharma, Thomas A. Paul

**Affiliations:** ^1^ Oncology Research Discovery Pfizer Worldwide Research and Development San Diego CA USA; ^2^ Arcus Biosciences Hayward CA USA

**Keywords:** drug resistance, epigenetic therapy, KDM1A, LSD1, small‐cell lung cancer

## Abstract

Small‐cell lung cancer (SCLC) is a heterogeneous disease, consisting of intratumoral and intertumoral neuroendocrine (ASCL1 and/or NEUROD1), mesenchymal‐like, and YAP‐driven transcriptional states. Lysine‐specific demethylase 1 (LSD1; also known as KDM1A) inhibitors have recently been progressed to clinical trials in SCLC based on a promising preclinical antitumor activity. A potential clinical limitation of LSD1 inhibitors is the heterogeneous drug responses that have been observed in SCLC cell lines and patient‐derived models. Based on these observations, we studied molecular and transcriptional signatures that predict patient response to this class of drug. Employing SCLC patient‐derived transcriptional signatures, we define that SCLC cell lines sensitive to LSD1 inhibitors are enriched in neuroendocrine transcriptional markers, whereas cell lines enriched in a mesenchymal‐like transcriptional program demonstrate intrinsic resistance to LSD1 inhibitors. We have identified a reversible, adaptive resistance mechanism to LSD1 inhibitors through epigenetic reprogramming to a TEAD4‐driven mesenchymal‐like state. Our data suggest that only a segment of SCLC patients, with a defined neuroendocrine differentiation state, will likely benefit from LSD1 inhibitors. It provides novel evidence for the selection of a TEAD4‐driven mesenchymal‐like subpopulation resistant to LSD1 inhibitors in SCLC patients that may require effective drug combinations to sustain effective clinical responses.

## Graphical abstract

Here, we identify intratumor and intertumor heterogeneity limits the activity of LSD1 inhibitors in SCLC. Activity of LSD1 inhibitors in SCLC is confided in cells enriched in neuroendocrine transcriptional programs. Transdifferentiation of SCLC into a TEAD4‐driven mesenchymal‐like state represents an acquired resistance mechanism to LSD1 inhibitors. Drug combinations targeting YAP‐TEAD pathways with LSD1 inhibitors may be an effective strategy to target intrinsic‐ and adaptive‐resistant populations and sustain effective clinical responses.

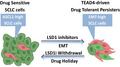

**Abbreviations**
ASCL1: achaete‐scute homolog 1
ATAC‐seq: assay for transposase‐accessible chromatin using sequencing
BRD4: bromodomain‐containing protein 4
CREBBP: cAMP response element‐binding protein‐binding protein
EMT: epithelial–mesenchymal transition
EP300: histone acetyltransferase p300 or E1A‐associated protein p300
EZH2: enhancer of zeste homolog 2
GESA: gene set enrichment analysis
GFI1B: growth factor independent 1B
GRP: gastrin‐releasing peptide
INSM1: insulinoma‐associated protein 1
LSD1/KDM1A: lysine‐specific histone demethylase 1A
MLL1/2: mixed lineage leukemia protein 1/2
NEUROD1: neurogenic differentiation 1
PDX: patient‐derived xenografts
RB1: retinoblastoma protein 1
scRNA‐seq: single cell RNA sequencing
SNAG: SNAIL/Gfi‐1 family zinc finger proteins
TEAD4: TEA domain family member 4
VIM: vimentin
ZEB1: zinc finger E‐box‐binding homeobox 1

## 1 Introduction

Small‐cell lung cancer (SCLC) is a neuroendocrine carcinoma that exhibits aggressive malignancy and a high propensity for early metastasis. In addition to common mutations in RB1 and TP53, molecular profiling studies have identified an epigenetic basis for SCLC disease initiation and progression featuring frequent mutations in chromatin‐modifying genes including CREBBP, EP300, and MLL1/2 as well as alterations in the distribution of DNA methylation [1]. Based on these observations, epigenetic drugs have been explored for therapeutic intervention to target tumor‐specific epigenetic vulnerabilities and reverse acquired epigenetic changes driving tumor growth and survival. Promising *in vitro* and *in vivo* preclinical data led to clinical trials exploring the use of histone deacetylase inhibitors including romidepsin or panobinostat in relapsed SCLC. Although both agents were well‐tolerated and demonstrated some evidence of tumor shrinkage and sustained stable disease, both clinical studies failed to demonstrate a significant benefit warranting continued development [2].

The emergence of a new generation of epigenetic drugs targeting specific transcriptional regulators of SCLC oncogenesis including BRD4, LSD1, and EZH2 has renewed interest in exploring epigenetic therapies in SCLC [3, 4, 5]. Among this class of new epigenetic drugs, inhibitors of the histone lysine demethylase LSD1 (KDM1A) have gained significant attention [6]. LSD1 is a member of the BRAF‐HDAC (BHC) corepressor complex shown to regulate expression of neuronal gene programs through interaction with REST [7, 8, 9, 10, 11, 12]. In addition to the previously reported antitumor activity in acute myeloid leukemia, LSD1 inhibitors have shown lineage‐specific activity in SCLC cell lines and patient‐derived xenograft models [5, 6, 13, 14, 15]. Although the mechanism of growth arrest conferred by these drugs is unclear, LSD1 has been shown to regulate neuroendocrine transcriptional programs critical for SCLC growth [5, 13]. Several recent reports have suggested that the primary mechanism of action of LSD1 inhibitors in both AML and SCLC occurs not only through catalytic inhibition of LSD1 enzymatic function but also through inhibition of the interaction of LSD1 with SNAG domain transcriptional factors such as INSM and GFI1B [14, 15]. Through these inhibitory mechanisms, LSD1 inhibitors can impact the expression of neuroendocrine lineage‐specific regulators such as ASCL1, required for SCLC lineage specification.

Recent studies have characterized SCLC as heterogeneous population, consisting of intratumoral and intertumoral neuroendocrine (ASCL1/NEUROD1), mesenchymal‐like, and YAP‐driven transcriptional states and subtypes [16, 17]. The particular SCLC subtypes may define vulnerabilities to therapeutic targets. In a breadth of efficacy screen, Mohammad *et al*. [5] identified only a subset of SCLC cell lines and primary samples with a DNA hypomethylation signature undergoing growth inhibition in response to GSK2879552. Similarly, in a panel of SCLC PDX tumors, Augert *et al*. [13] identified selective activity of ORY‐1001 only in models capable of NOTCH pathway activation. Moreover, many sensitive SCLC cell lines and primary tumor models display only partial responses to LSD1 inhibition even after long durations of treatment. The recent advancement of both irreversible and reversible LSD1 inhibitors into SCLC clinical trials warrants the study of potential drug resistance mechanisms that may prevent patients from responding to this class of drugs.

In this study, we have addressed potential intrinsic and acquired drug resistance mechanisms to LSD1 inhibitors in SCLC. Utilizing a recently identified gene coexpression network defining SCLC neuroendocrine and mesenchymal states [18, 19], we have identified that sensitivity to LSD1 inhibitors in SCLC is confined primarily to cell lines that express neuroendocrine transcriptional programs. Heterogeneous drug responses in SCLC cell lines reflect a preexisting cell intrinsic drug resistance mechanism enriched in cell lines expressing mesenchymal‐like transcriptional programs. Using single cell RNA‐seq and ATAC‐seq, we demonstrate that continuous treatment with LSD1 inhibitors results in the emergence of drug‐tolerant subclones with a *de novo* mesenchymal‐like transcriptional state driven through a TEAD4 transcription factor program. We also highlight that acquired resistance to LSD1 inhibitors appears as an ‘epi‐stable’ state. Under drug withdrawal, drug‐tolerant SCLC cells transition between mesenchymal and neuroendocrine phenotypes and regain sensitivity to the drug. Collectively our data provide novel insight in the mechanism contributing to heterogeneous responsiveness of SCLC to LSD1 inhibitors, and selection of mesenchymal‐like enriched subclones is likely to present a barrier to effective single‐agent responses in the clinic.

## 2 Materials and methods

### 2.1 Cell lines and reagents

Human SCLC cell lines used in this study were obtained from the American Type Culture Collection (ATCC, Manassas, VA, USA) , Sigma‐Aldrich (St. Louis, MO, USA), or Deutsche Sammlung von Mikroorganismen und Zellkulturen (DSMZ, Brunswick, Germany) and grown in manufacturer’s specified growth medium and environmental conditions. Cell growth medium was purchased from Life Technologies (Carlsbad, CA, USA) or Lonza (Basel, Switzerland).

GSK690 [20] and OG‐86 [21] were synthesized by WuXi AppTec (Shanghai, China) using previously disclosed structures. Structures were confirmed using NMR (nuclear magnetic resonance spectroscopy) and LCMS (liquid chromatography–mass spectrometry). Compounds were solvated in DMSO (Sigma, St. Louis, MO, USA) at 30 mm for use in *in vitro* experiments.

### 2.2 Human lung cancer tissue microarray

Lung cancer tumor microarray (14–140) was purchased from US Biomax, Inc. (Derwood, MD, USA) Immunohistochemical staining and evaluation were performed on a single TMA slide containing 200 microarray dot tissue samples representing a broad array of human lung cancer histologic subtypes as well the inclusion of 20 normal lung tissue samples for comparison. Immunohistochemical procedure and staining were developed and optimized with known LSD1‐positive control tissues to ensure proper specificity. Pathologist scoring method for all evaluated TMA cores was semiquantitative and based on a range of tumor cell positivity corresponding to each score, with a score of 1 indicating rare positivity, 2 showing 3–10% positivity, 3 showing 11–30% positivity, and 4 for everything greater than 30.

Immunohistochemical‐stained slides were prepared using a Leica Bond automated staining system. Briefly, slides underwent antigen retrieval (Leica ER2, Wetzlar, Germany) for 20 min followed by incubation with rabbit anti‐LSD1 1 : 100 (Cell Signaling Technology, Danvers, MA, USA, #2139) for 40 min at room temperature. Bond Polymer Refine (Buffalo Grove, IL, USA, Bond #DS9800) kit with poly‐HRP and 3,3‐diaminobenzidine tetrahydrochloride (DAB substrate chromogen) was used to detect and visualize the LSD1 staining. Finally, the slides were counterstained with hematoxylin, dehydrated, and mounted. The study methodologies conformed to the standards set by the Declaration of Helsinki and were approved by Pfizer ethics committee.

### 2.3 Preparation of cell extracts and western blot analysis

Adherent and suspension cell populations were collected and washed in PBS prior to lysis in RIPA buffer (Sigma) with protease inhibitor cocktails (Roche, South San Francisco, CA, USA) plus PMSF. Cell lysates were briefly sonicated prior and precleared by centrifugation. 30 µg of protein was loaded on 4–12% SDS/PAGE and transferred to nitrocellulose membranes. Samples were incubated overnight at 4 °C in primary antibodies and 1 h at room temperature in secondary antibodies. Imaging was performed using LiCor Imaging System (Lincoln, NE, USA).

Antibodies used in this study are KDM1A (Millipore cs207350, Bethyl, Montgomery, TX, USA, A300‐215A), NSE (CST, Danvers, MA, USA, 9536), SYP (CST 12270), GRP (Sigma HPA007314), NCAM (CST 3576), CHGA (Abcam, Branford, CT, USA, 45179), FOXA2 (CST 3143), OVOL2 (Abcam 129161), LEF1 (CST 2286), ASCL1 (Abnova, Walnut, CA, USA, H0429‐M02), SOX2 (CST 4900), INSM1 (Abcam 170876), NEUROD1 (117562), SMAD3 (CST 9513), NFKB2 (CST 3017), CMYC (CST 9402), MITF (Abcam 140606), SNAIL (CST 3895), VIM (CST 5741), CDH2 (CST 14215), CDH1 (CST 3195P), and ZEB1 (CST 3396P).

### 2.4 Long‐term cell proliferation assays

For drug sensitivity studies, cell lines were plated in triplicate 10‐cm^2^ dishes at drug concentrations of 1000, 300, 100, 30, 10, 3, and 1 nm in DMSO. Every 3 or 4 days, adherent and suspension cells were collected and 2 mL of cells was transferred to a new 10‐cm^2^ dishes for subsequent culture. 8 mL of fresh cell culture medium was added to each plate, and drug was added at proper concentrations to each dish. Three aliquots of 1 mL of cells were collected in 1.5‐mL microcentrifuge tube, concentrated by centrifugation to a 100 µL volume, and added to a 96‐well plate. Relative cell numbers were determined either by cell counting of three independent aliquots using a hemocytometer or by CellTiter‐Glo reagent (Promega, Madison, WI, USA).

### 2.5 RNA‐seq and expression profiling experiments

RNA‐seq was accomplished on SCLC cell lines treated in triplicate with DMSO or 0.3 µm GSK690 for either 3 or 10 days. RNA‐seq libraries were generated following manufacture’s protocols and sequenced paired‐end on the HiSeq 2000 at read length 50 or 100 nt (only for COR‐L88). FastQ data generated by HiSeq were QCed and trimmed before being aligned to the HG19 human reference genome and then quantified using the rsem package [22] The RNA‐seq DE (differential expression) analysis was done by using the deseq r package after rsem [23]. For this analysis, the program used raw counts as input and ran its normalization method using a scaling factor computed as the median of the ratio, for each gene, of its read count over its geometric mean across all lanes.

The SCLC cell line mRNA expression data were downloaded from CCLE 19Q1 [24]. For differential expression analysis, genes that are not expressed (TPM < 3 in all cell lines) are first removed, and moderated *t*‐test was conducted using limma package with Empirical Bayes shrinkage [25] in sensitive (*n* = 10) vs resistant (*n* = 12) cell lines using log2‐TPM values with FDR multiple testing correction. Differential expression cutoff is set at FDR < 5% and FC > 2. The heatmaps were generated using the complex heatmap package in r [26]. To identify enriched pathways associated with LSD1 sensitivity, we calculated a significance score defined as follows: −log2(FDR) X (difference in mean TPM between sensitive and resistant lines) and loaded this score into GSEA prerank for pathway analysis [27]. The neuroendocrine (NE) and mesenchymal‐like (ML) network signature genes for 53 SCLC CCLE cell lines were defined as previously described [19, 28]. For overlapping analysis between differential genes and NE/ML network genes, hypergeometric test was performed between each gene set, and the median TPM value of NE or ML module genes was plotted with boxplot with two‐sample *t*‐test on sensitive and resistant cell lines.

For TCGA SCLC tumor gene expression analysis, the 2015 UCologne RNA‐seq *z*‐score data were downloaded from cBio portal (https://www.cbioportal.org/). Genes containing NA readings were removed, and duplicated gene names were merged by their mean expression *z*‐score. This resulted in a total of 18 598 genes in 81 patient tumors. To calculate gene signature scores, the median expression *z*‐score from each signature was calculated for each tumor. The gene expression heatmap was generated using the complex heatmap package in r [26].

### 2.6 Single cell isolation and scRNA‐seq

NCI‐H69 cells were treated in duplicate with DMSO or 0.3 μm GSK690 for 21 days. The single cell suspension was isolated by passing the harvested cells through a 40‐μm Flowmi^TM^ tip strainer followed by incubation with 0.25% Trypsin‐EDTA in ice for 10 min and at 37 °C for 10 min to get a final concentration of 3 × 10^5^ cells·mL^−1^. The single cells were then processed through the Chromium 3′ Single Cell Platform using v2 reagents (10X Genomics, Pleasanton, CA, USA) per the manufacturer’s protocol. In brief, a total of 6536 single cells were detected with 3603 cells analyzed in the DMSO vehicle group and 2903 in LSD1 inhibitor‐treated group. The cells were partitioned into Gel Beads in Emulsion in the Chromium instrument, where cell lysis and barcoded reverse transcription of RNA occurred, followed by amplification, shearing, and 5′ adaptor and sample index attachment. Libraries were sequenced on an Illumina HiSeq 4000 (San Diego, CA, USA).

For single cell RNA‐seq analysis, gene‐cell count matrix was generated with Cellranger pipeline (10X Genomics, Pleasanton, CA, USA). All downstream analyses, including cell filtering, data normalization, and cell clustering, were performed using the r seurat v3 package [29]. In brief, to remove low‐quality data, cells that have unique gene counts between 200 and 6000 and mitochondrial reads < 10% were kept for further analysis. Read counts were then normalized for total counts in each cell, scaled to 10 000, and then log‐transformed. To remove confounding factors associated with assay quality and cell cycle stages, we used SCTransform [30] to regress out percent mitochondrial reads, percent ribosomal reads, and cell cycle S/G2M score in each single cell. Top 3000 variable genes were selected for downstream analysis. To facilitate single cell comparison between control and LSD1 inhibitor treatment, we used Harmony package [31] with default settings to integrate single cell data and used UMAP [32] for two‐dimensional data embedding. Differentially expressed genes between each cluster or control vs drug‐treated cells were called using the Seurat ‘FindMarkers’ function with these parameters: min.pct = 0.2, logfc. threshold = 0.25, with a cutoff of FDR < 5%. Gene expression values or scores in UMAP were capped at 5% and 95% quantile to eliminate outlier bias during data representation.

### 2.7 ATAC‐seq and data analysis

NCI‐H69 cells were treated in triplicate with DMSO or 0.3 μm GSK690 for 21 days. The transposition assay was performed. Briefly, 50 000 nuclei from each sample were used in each reaction with 20 µL of transposition mix and incubated at 37 °C for 60 min. qPCR was performed, and eight cycles were performed. The library was purified with Ampure XP beads (A63880, Beckman Coulter, Brea, CA, USA), quantified using Qubit (Qubit dsDNA HS Assay Kit, Q32851, Thermo Fisher Scientific, San Diego, CA, USA), and checked for size distribution using 4200 TapeStation (High Sensitivity D1000 ScreenTape, 5067‐5584, Agilent Technologies, Santa Clara, CA, USA). Sequencing was performed with Illumina HiSeq4000 (San Diego, CA, USA) (50 bp PE, Nextera v2 libraries).

For ATAC‐seq data analysis, paired‐end sequencing reads for each sample were mapped to hg38 human reference genome with Bowtie 2 [33] using the following parameters: ‐t ‐X 2000 ‐‐no‐mixed ‐‐no‐discordant. Sam files were converted into Bam with samtools. Peak calling was performed with MACS2 [34] using these parameters: callpeak ‐g hs ‐‐nolambda ‐‐nomodel ‐‐shift ‐100 ‐‐extsize 200 ‐‐bdg ‐‐SPMR ‐q 5e‐2. Peaks overlap with ENCODE black list regions were removed from downstream analysis using bedtools. BedGraph files were converted to Bigwig using bedgraphtobigwig [35], and signal tracks were visualized with IGV [36]. To correct for ATAC‐seq bias, we extracted genome‐wide signal at 100‐bp resolution using deeptools [37] and performed quantile normalization on the signal data using the r package preprocesscore (https://rdrr.io/bioc/preprocessCore/). Differential peak calling between the control and drug‐treated samples was performed with DESeq2 [38], using merged peaks between all samples (total 250 646 peaks). Significant differential peaks were defined as follows: FDR < 0.01 & fold change > 2.5, and the mean read count for each peak is > 40% quantile of all mean read count (to remove low signal regions). This results in 548 open peaks and 838 closed peaks after drug treatment. GREAT analysis [39] was performed on the differential peaks to identify pathways associated with the drug treatment. To predict transcription factor (TF) binding perturbed by the drug treatment, we employed chromvar package [40] with the ‘human_pwms_v2 motif’ database to identify TF binding DNA motifs that are significantly open or closed upon treatment. *t*‐Test with FDR correction was performed on all TF *z*‐scores to identify significant TF motifs.

## 3 Results

### 3.1 LSD1 is highly expressed in small‐cell lung cancer relative to other lung cancer subtypes

**Fig. 1:**
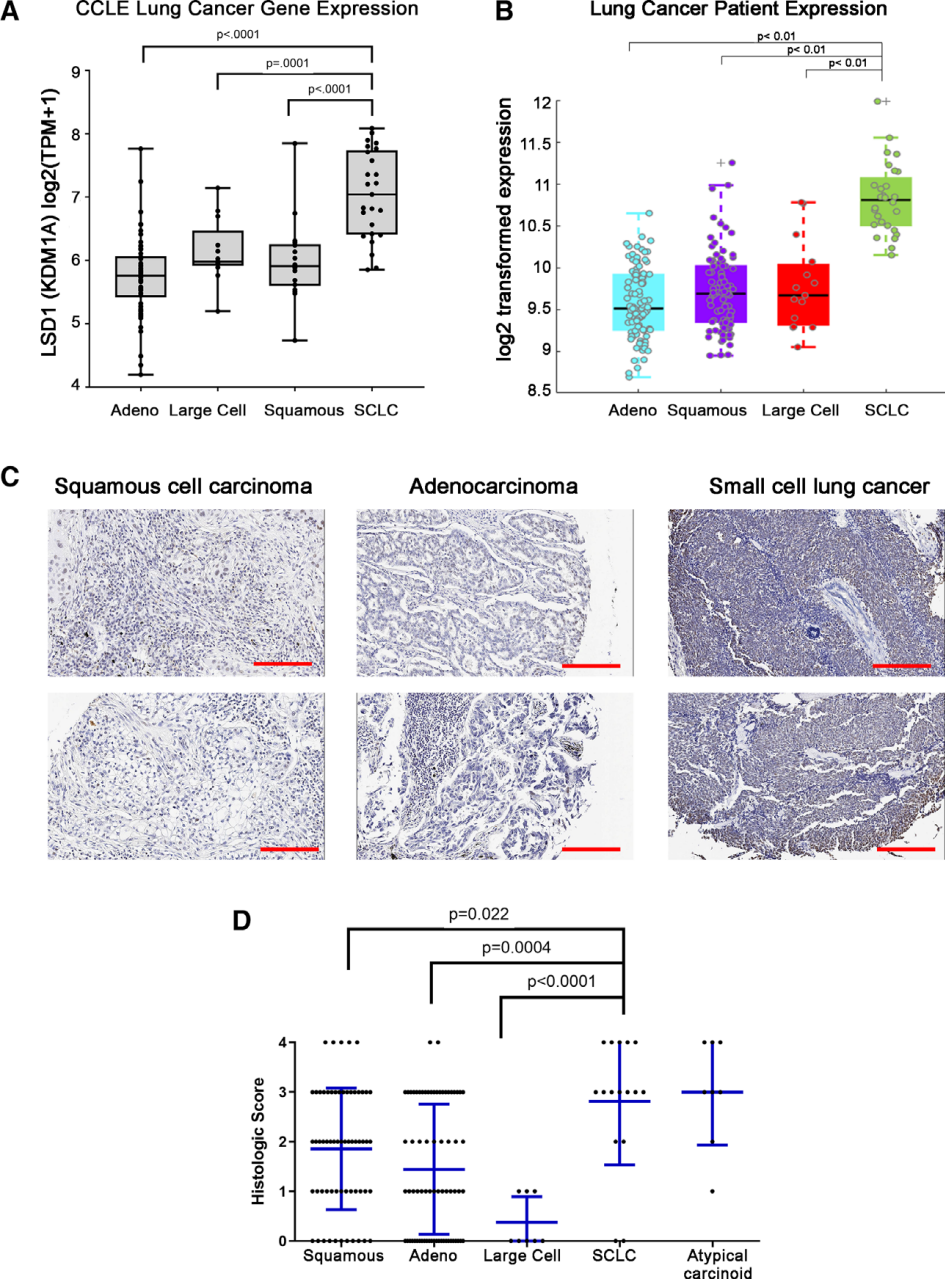
LSD1 is highly expressed in small‐cell lung cancer relative to other lung cancer subtypes. (A) Expression of LSD1 (KDM1A) mRNA from *n* = 185 lung cancer cell lines derived from the CCLE dataset at the Broad Institute. (B) LSD1 expression data in 261 primary lung tumors from Clinical Lung Cancer Genome Project (CLCGP). (C) Representative images of LSD1 expression with scale bar at 250 µm by immunohistochemistry (IHC) in *n* = 200 lung cancer tissue microarray. (D) Quantitation of LSD1 expression in *n* = 200 microarray dot tissue samples all from a broad array of human lung cancer histologic subtypes and including *n* = 20 normal lung samples for comparison. LSD1 IHC immunoreactivity was given a histologic semiquantitative score (0–4; 4 being highest intensity staining) for each sample. Data presented represent mean ± 1 standard deviation (SD). Statistical significance was determined by one‐way ANOVA with Tukey HSD *post hoc* test for (A), (B), and (D).

Overexpression of chromatin modifiers has been shown to influence their function in cancer [41]. In lung cancer, LSD1 mRNA expression is highly expressed in SCLC relative to other lung cancer cell lines (Fig. 1A). Expression datasets from Clinical Lung Cancer Genome Project (CLCGP) [42] confirm LSD1 is also expressed highly relative to other lung cancer subtypes in primary patient samples (Fig. 1B). To further confirm LSD1 expression levels at the protein level, we conducted immunohistochemistry (IHC) on a lung cancer tissue microarray. IHC staining confirmed a statistically significantly higher staining intensity in SCLC specimens relative to that of squamous cell carcinoma, adenocarcinoma, and large cell carcinoma (1‐way ANOVA Fig. 1C,D).

### 3.2 SCLC cell lines show heterogeneous responses to LSD1 inhibitors

Resulting from the observation of high expression of LSD1 in SCLC, we assessed whether SCLC cell lines were dependent on LSD1 activity for their growth. Using two shRNAs targeting LSD1, we observed growth inhibition in the NCI‐H526 cell line that became strongest after 14 days of target knockdown (Fig. S1A). Interestingly, DMS‐114 cells did not appear sensitive to LSD1 knockdown over 14 days even though there was a significant loss of LSD1 protein level by western blot (Fig. S1B).

Several reports have recently described sensitivity in SCLC cell lines to LSD1 inhibitors. To study this further, we profiled drug sensitivity to the FAD reversible inhibitor GSK690 (Ki = 4 nm) [20] as well as the FAD irreversible compound OG‐86 (IC50 = 47 nm) [21] in a panel of SCLC cell lines (Fig. S2). Due to the delayed onset of proliferation changes observed in shRNA experiments, we tested the effects of GSK690 and OG‐86 on SCLC cell line growth in long‐term proliferation assays of 17–21 days. Consistent with shRNA results, we observed differential responses in many SCLC cell lines. In NCI‐H1417, NCI‐H187, and NCI‐H889 cells, dose‐dependent growth inhibition was initially observed at 7–10 days of both GSK690 and OG‐86 treatments with continued effects observed until days 17–21 (Fig. S3A–C). DMS‐114 cells, which were insensitive to LSD1 shRNA knockdown, were also insensitive to both GSK690 and OG‐86 up to 17 days of treatment at concentrations up to 1.0 µm OG‐86 and 1.0 µm GSK690 (Fig. S3D). Treatment with LSD1 inhibitors resulted in cytostatic responses in sensitive SCLC cell lines. In COR‐L88 and NCI‐H1417 cells, an increase in sub‐G1 phase was observed upon 7 days of GSK690 treatment (Fig. S3E) along with an increase in cells in G1 phase (42% G1 for 1 µm GSK690 vs 25% G1 for DMSO in COR‐L88 cells; Fig. S3F). In contrast, GSK690 treatment in NCI‐H526 arrested cell growth without obvious accumulation in any stage of the cell cycle (Fig. S3E). Interestingly, the antiproliferative effects of LSD1 inhibitors plateau in their percent inhibition even after 17–21 days of continuous drug treatment due to the emergence of a subpopulation of slowly proliferative persister cells in NCI‐H1417 and NCI‐H187 cells (Fig. S3). These data suggest that SCLC cell lines possess both intrinsic and acquired resistance mechanisms to LSD1 inhibitors.

### 3.3 Neuroendocrine and mesenchymal expression signatures stratify sensitivity to LSD1 inhibition

**Fig. 2:**
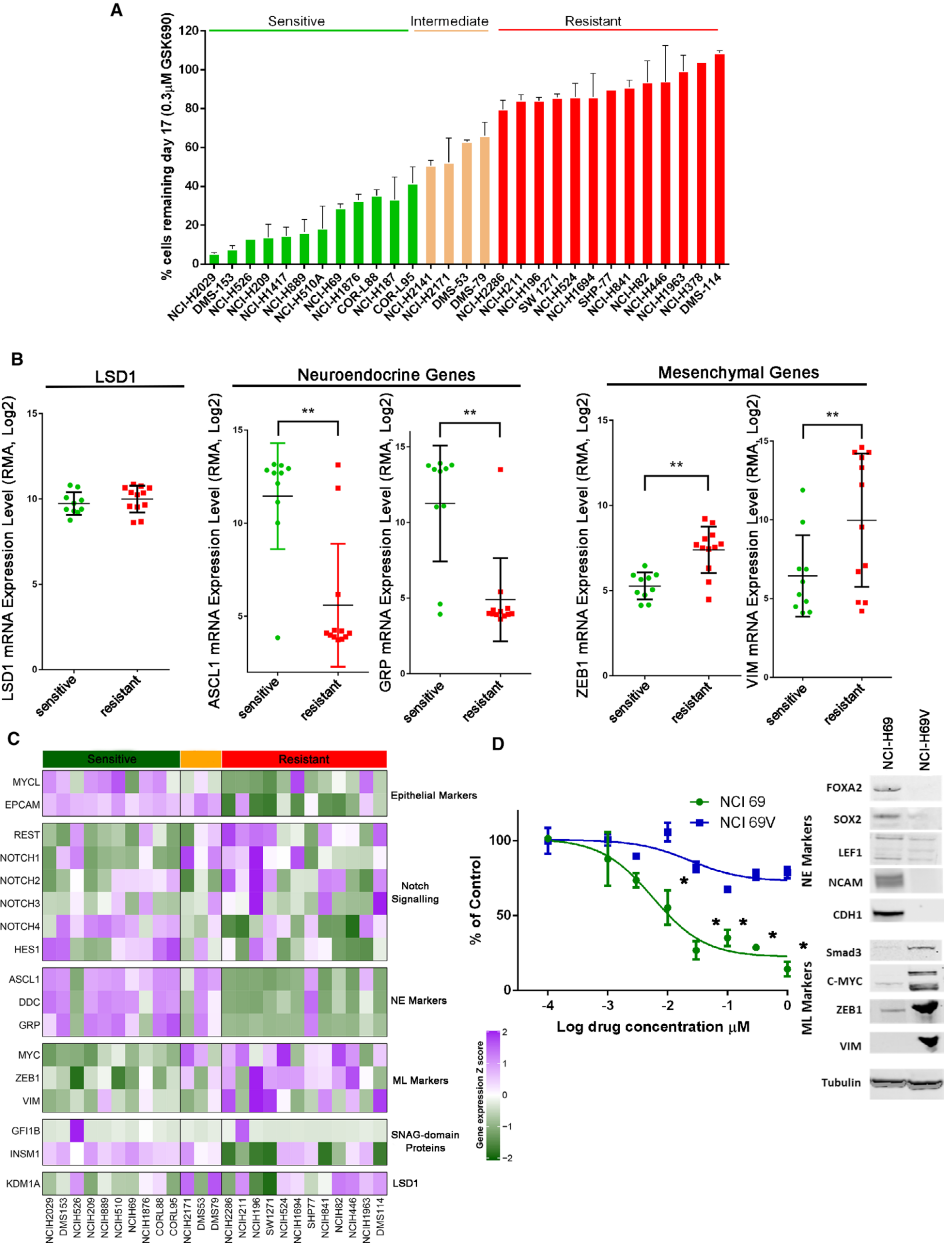
Differential sensitivity of SCLC cell lines to LSD1 inhibitors stratifies with expression of neuroendocrine markers. (A) SCLC cell sensitivity to 0.3 µm GSK690 at day 17 based on the percentage of remaining cells relative to DMSO control. Long‐term proliferation assays were run with *n* = 3 biologically independent replicates presented with mean ± SD. Sensitive cell lines are reflected by > 50% growth inhibition, resistant cell lines at < 20% growth inhibition. (B) Expression of LSD1, GRP, ASCL1, VIM, and ZEB1 in GSK690‐sensitive and GSK690‐resistant cell lines based on CCLE mRNA expression data, *n* = 2 independent experiments performed ± SD. Two sample *t*‐test **P‐values < 0.05 are shown. (C) Heatmap of CCLE mRNA expression of specific marker genes defining SCLC subtypes. Gene expression values are shown as row normalized *z*‐scores from log2(TPM+1). (D) GSK690 dose response in NCI‐H69 compared with NCI‐H69V cells at day 17 in the mean of *n* = 2 independent experiments performed in quadruplets ± SD. Two‐way ANOVA multiple comparisons with *P‐values < 0.001 are shown. Western blot using indicated antibodies demonstrating protein expression of neuroendocrine (NE) and mesenchymal (ML) markers in NCI‐H69 and NCI‐H69V cells.

Across the SCLC cell line panel, 12 out of 29 tested SCLC cell lines showed > 50% growth inhibition in response to 0.3 µm GSK690 treatment at day 17 (Fig. 2A). A nonenzymatic mechanism of action has recently been proposed to account for the antitumor activity of LSD1 inhibitors in SCLC [15]. In our cell panel, we identified that all SCLC cell lines sensitive to GSK690 express SNAG domain proteins INSM1 or GFI1B; however, we also observed examples of INSM1 or GFI1B‐expressing cell lines that were also resistant to the drug (Fig. 2C). Thus, the expression of SNAG domain proteins alone is insufficient to define cell lines that will respond to GSK690.

To identify additional biomarkers that predict sensitivity to GSK690, we explored both mutational and gene expression features of SCLC cell lines from available CCLE data [24] LSD1 expression level alone did not correlate with drug sensitivity (Fig. 2B,C). Additionally, using both univariate and multivariate association methods, no mutation significantly correlated with cell line sensitivity (data not shown). Analysis of the cell line gene expression by principal component analysis suggests that cell lines sensitive to GSK690 stratify in a group with distinct transcriptional state compared to insensitive cell lines with exception of NCI‐H526 and NCI‐H1963 cells that appear to be outliers (Fig. S4A). SCLC cell lines can be stratified by neuroendocrine/epithelial (NE) and mesenchymal‐like (ML) features. Using established gene expression markers of these SCLC cell states [16, 17], we found enrichment in GSK690‐sensitive cell lines in expression of epithelial genes, MYCL and EPCAM, and neuroendocrine genes, GRP, DDC, and ASCL1 (Fig. 2B,C and Fig. S5). SCLC cell lines resistant to GSK690 featured higher expression of mesenchymal genes MYC, ZEB1, and VIM (Fig. 2B,C and Fig. S5). These data suggest that neuroendocrine‐like SCLC cell lines may be more sensitive to LSD1 inhibition while mesenchymal‐like SCLC states may confer resistance to LSD1 inhibitors.

To experimentally address the intrinsic drug resistance of mesenchymal‐shifted SCLC cells to LSD1 inhibitors, we treated a mesenchymal variant form of NCI‐H69 cells, NCI‐H69V [43] with GSK690. NCI‐H69 cells grow in suspension and show enrichment in NE transcription factors FOXA2, SOX2, LEF1, NCAM, and E‐cadherin (Fig. 2D). In contrast, NCI‐H69V cells grow as adherent cells and express a ML signature‐enriched transcription factors SMAD3, MYC, ZEB1, and VIM [19] (Fig. 2D). In line with our hypothesis, mesenchymal‐shifted NCI‐H69V appeared to be resistant to GSK690 treatment, showing only 22% growth inhibition compared with 85% growth inhibition observed in parental NCI‐H69 cells (Fig. 2D).

**Fig. 3:**
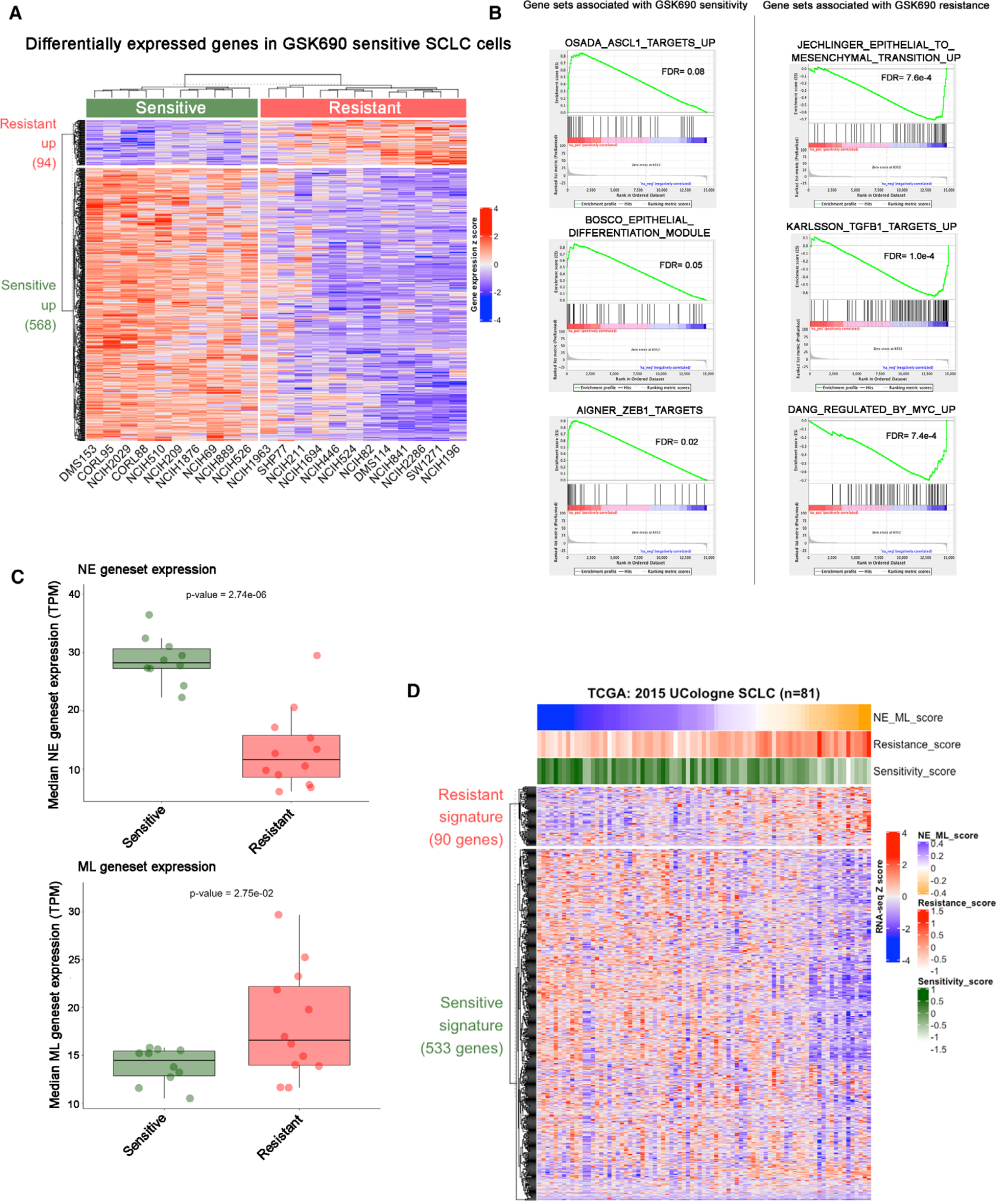
Connection of LSD1 sensitivity signature with neuroendocrine and mesenchymal‐like network signatures. (A) CCLE gene expression heatmap with hierarchical clustering of differentially expressed (DE) genes between sensitive (*n* = 10) and resistant (*n* = 12) cell lines. Gene expression values are shown as row *z*‐score. DE gene cutoff is FDR < 5% and FC > 2. (B) Gene Set Enrichment Analysis (GSEA) of differential gene expression between sensitive and resistant cell lines from (A). (C) Boxplot depicting the CCLE median expression values (TPM) of previously published neuroendocrine (NE) and mesenchymal‐like (ML) signatures [19] in sensitive and resistant cell lines from (A). Two sample *t*‐test *P*‐values are shown. (D) Heatmap representation of RNA‐seq *z*‐score for TCGA 2015 UCologne SCLC data. The column represents 81 SCLC patient tumors, and the row represents LSD1i signature genes generated from (A). Genes captured for each signature from the TCGA samples were as follows (captured genes/total signature genes): 3426/3471 for NE signature; 1169/1179 for ML signature; 533/568 for LSD1i sensitivity signature; 90/94 for resistance signature. The NE‐ML score was calculated by subtracting the NE score from the ML score for each tumor. Hypergeometric test *P*‐values are shown.

To further refine the gene expression signature associated with LSD1 inhibitor resistance, we performed differential expression analysis between LSD1 inhibition sensitive and resistant SCLC cells from CCLE cell line data. Using this approach, we identified a differential gene expression signature (FDR < 5% and FC > 2) stratifying cell lines sensitive and resistant to LSD1 inhibitors (Fig. 3A). By performing Gene Set Enrichment Analysis (GSEA) [27] on these differential genes, we found that gene sets of ASCL1 targets, epithelial differentiation, and ZEB1 repressive sites are upregulated in the sensitive cells, whereas EMT, TGF‐beta, and MYC pathway gene sets are significantly upregulated in the resistant cells (Fig. 3B).

Since sensitivity to LSD1 inhibition was associated with the presence or absence of expression of canonical neuroendocrine and mesenchymal markers in our previous analysis, we compared the GSK690 differentially expressed gene signature to an established gene coexpression network that delineates tumor heterogeneity observed in SCLC tumor samples as neuroendocrine (NE) and mesenchymal‐like (ML) [18, 19]. Aligning the LSD1 inhibition differential expression signature to genes defining the NE (*n* = 1102) or ML (*n* = 2663) coexpression networks [19], we found that sensitive cell lines significantly upregulated NE network genes, while resistant lines significantly upregulated ML network genes (Fig. 3C). In addition, genes upregulated in LSD1 inhibitor sensitive cell lines significantly overlapped with NE network genes while genes upregulated in LSD1 inhibitor‐resistant cell lines significantly overlapped with the ML network genes (Fig. S4B). Overall, these data highlight the context specificity of GSK690 activity in neuroendocrine SCLC subtypes and explain the heterogeneous drug responses observed in SCLC cell lines.

We next assessed the potential of the LSD1 sensitivity signature in identification of SCLC patients that might be sensitive or resistant to LSD1 inhibition. Using the differential gene expression signatures that stratify cell lines sensitive and resistant to LSD1 inhibitors (Fig. 3A), TCGA 2015 UCologne SCLC patient dataset was analyzed. Patients with high expression of NE genes correlated with genes increased in expression in the LSD1 sensitivity signature score. Conversely, patients enriched in ML signature overlapped with LSD1 inhibitor resistance gene expression (Fig. 3D). Thus, the LSD1 inhibitor gene expression signature defined distinct subsets of SCLC patients that are predicted to be either sensitive or resistant to LSD1 inhibitors.

### 3.4 Drug‐tolerant cells with mesenchymal‐like states emerge after LSD1 inhibitor treatment

**Fig. 4:**
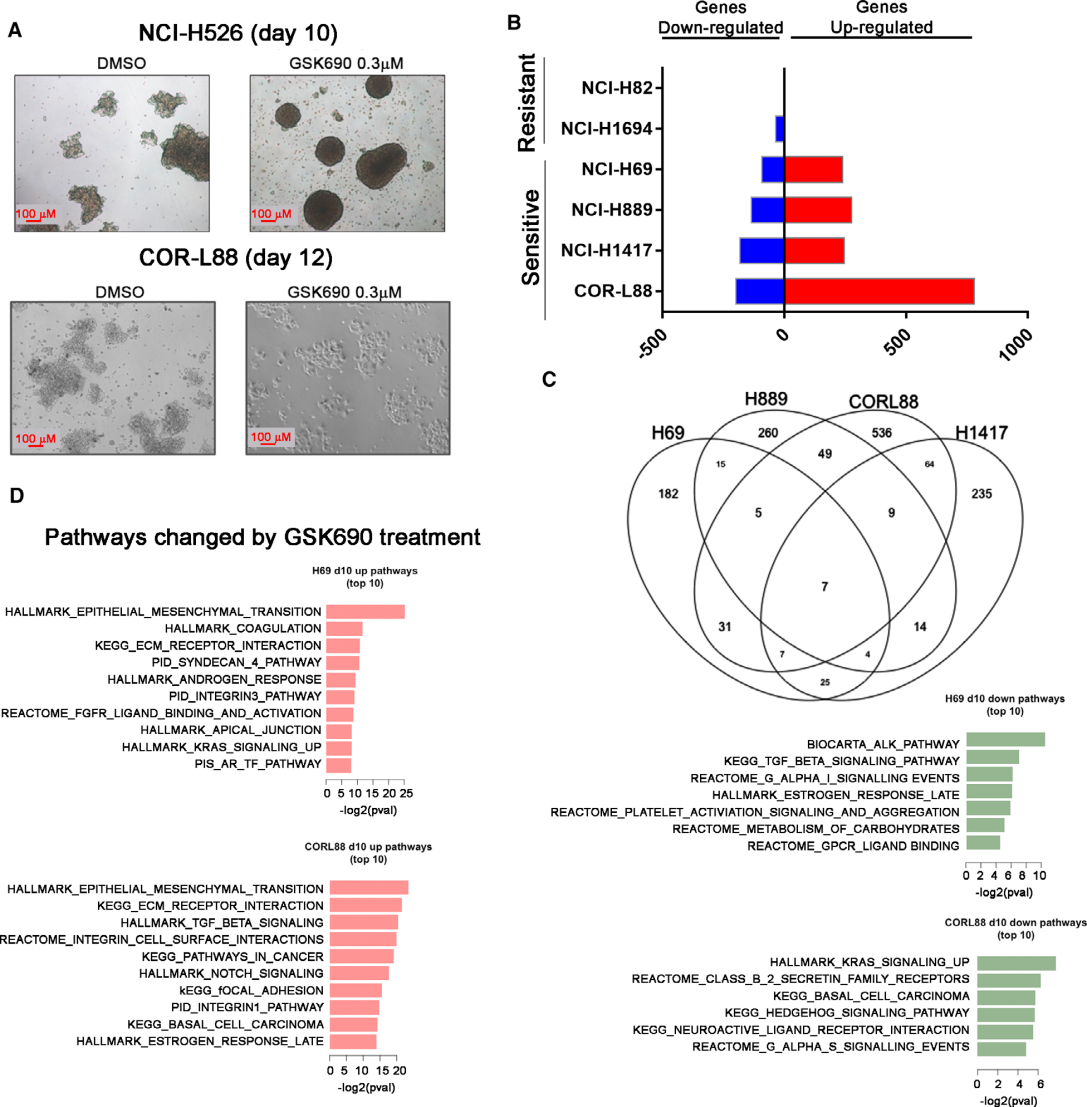
LSD1 inhibitor treatment results in gene expression changes in axonal and EMT pathways (A) Microscope images of NCI‐H526 and COR‐L88 cell clusters (scale bar, 100 µm) grown in tissue culture following treatment with DMSO or 0.3 µm GSK690 for indicated times with *n* = 3 independent experiments. (B) RNA‐seq analysis of genes significantly altered (FDR < 0.05, fold change ≥ 2) in expression in GSK690‐sensitive cell lines NCI‐H69, NCI‐H1417, NCI‐H889, and COR‐L88 and GSK690‐insensitive cell lines NCI‐H82 and NCI‐H1694‐treated with vehicle (DMSO) or 0.3 µm GSK690 for 10 days, with *n* = 3 biologically independent replicates. (C) Venn diagrams reflecting overlap of genes significantly upregulated or downregulated by GSK690 treatment between SCLC cell lines. (D) Pathway analysis of genes altered in expression (FDR < 0.05, fold change ≥ 2) by GSK690 treatment utilizing MSigDB pathway databases. Top 10 enriched pathways are shown with the *P*‐value cutoff of 0.05.

Small‐cell lung cancer cell lines grow in floating cell aggregates; however, a subset of cell lines will grow attached to tissue culture plastic [44, 45]. Cell lines which grow in suspension tend to express neuroendocrine markers while adherently growing cell lines are enriched in mesenchymal biomarkers [19]. SCLC cells that persist after day 10 of 0.3 µm GSK690 treatment show profound morphologic changes. NCI‐H526 cells changed from loose floating aggregate to tightly adherent spheres (Fig. 4A). COR‐L88 cells changed morphologically and gained adherence to tissue culture‐treated plates at day 12 of GSK690 treatment (Fig. 4A).

Since morphological changes are known to occur in SCLC cells that transition to a mesenchymal state [46], we assessed whether LSD1 inhibitor persister populations shift to a mesenchymal differentiation state. We performed bulk RNA‐seq on four sensitive cell lines (NCI‐H889, NCI‐H1417, NCI‐H69, and COR‐L88) and two insensitive cell lines (NCI‐H82 and NCI‐H1694). At day 10 of GSK690 treatment, a larger number of genes show changes in all four sensitive cell lines, with the majority of genes showing upregulation (FDR < 0.05, fold change ≥ 2) (Fig. 4B). Interestingly, only five genes and 43 genes were significantly altered after 10 days of GSK690 treatment in two GSK690‐insensitive models, NCI‐H82 and NCI‐H1694, respectively (Fig. 4B). Evaluation of common transcriptional changes occurring in sensitive models revealed only seven genes as consistently altered (FDR < 0.05, fold change ≥ 2) in expression at day 10 in all four cells lines (Fig. 4C). However, the data showed more convergence at a pathway level with several common pathways identified showing consistent changes due to GSK690 treatment in the four sensitive cell lines. Pathways associated with genes upregulated by GSK690 treatment are enrichment in epithelial–mesenchymal transition pathway, TGF‐beta signaling, NOTCH signaling, and focal adhesion‐related signaling consistent with the observed changes in adhesion (Fig. 4D, Fig. S4C). Conversely, pathways enriched in genes downregulated by GSK690 treatment did not show overlap between different cell lines and include various signaling pathways (Fig. 4D, Fig. S4C).

Changes in neuroendocrine and mesenchymal markers were also observed at the protein level in GSK690‐sensitive cell models (Fig. S6). COR‐L88 cells showed the strongest shift into a mesenchymal‐like state showing downregulation of neuroendocrine proteins NSE, GRP, NCAM, and CHGA and neuroendocrine transcription factors FOXA2, ASCL1, and SOX2. COR‐L88 cells also showed strong upregulation of the mesenchymal protein VIM. Other GSK690‐sensitive cell models showed only small changes in protein levels across our panel with GRP reduction and ZEB1 and CDH2 upregulation being the most consistent markers of response (Fig. S6). We observed no consistent changes in the protein levels of any neuroendocrine and mesenchymal markers in GSK690‐insensitive cell lines. Importantly, the extent of mesenchymal gene induction in GSK690‐treated SCLC cells does appear comparatively lower relative to what we observed in mesenchymal‐shifted NCI‐H69V cells and thus may represent a ‘partial EMT’ [47].

### 3.5 LSD1 inhibitor persister cells evolve from epigenetically distinct subpopulations

**Fig. 5:**
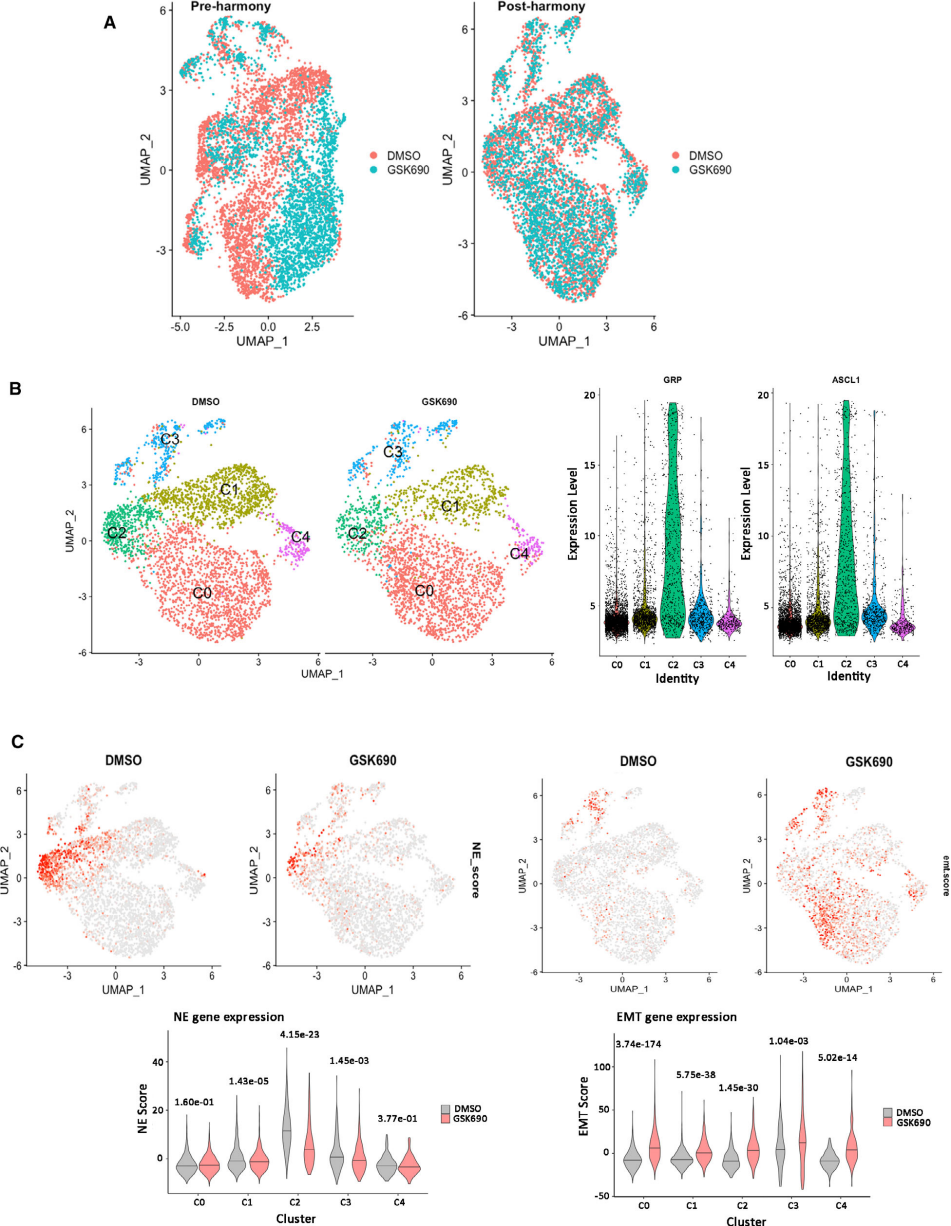
Single cell RNA‐seq reveals emergence of cell subpopulations enriched in EMT pathways following LSD1 inhibitor treatment (A) Uniform Manifold Approximation and Projection (UMAP) representation of pre‐ and post‐Harmony integrated single cell RNA‐seq data following treatment with vehicle (DMSO) or 0.3 μm GSK690 for 21 days. (B) UMAP representation of cell clustering and GRP and ASCL1 expression violin plot in each cluster. (C) Mean expression of NE or ML gene sets in UMAP or as violin plots in each cluster (*n* = 3 biologically independent replicates). Two sample *t*‐test *P*‐values are shown.

To define the origin of mesenchymal‐like persister cells, we conducted single cell RNA‐seq analysis on NCI‐H69 cells treated with DMSO or 0.3 μm GSK690 for 21 days. Harmony [31] was applied to integrate DMSO and treatment groups so that gene expression within each single cell subpopulation can be directly compared with obtain subpopulation‐specific differentially expressed (DE) genes after treatment (Fig. 5A). To identify distinct cell subpopulations, unsupervised cell clustering was performed and five clusters were selected manually after visualization using uniform manifold approximation and projection (UMAP) (Fig. 5B and Fig. S7A). Cell subpopulations in Clusters 0 and 3 were increased in 0.3 μm GSK690‐treated group relative to the DMSO group (Fig. S7A). In contrast, cell subpopulations in Clusters 1 and 2 in DMSO group were reduced after 21 days of LSD1 inhibitor treatment (Fig. S7A). There was no significant difference in the Cluster 4 cell subpopulation between DMSO and treated groups. Cluster 2 was identified as a neuroendocrine‐like subpopulation, as neuroendocrine transcriptional factors GRP and ASCL1 were specifically upregulated in this subpopulation (Fig. 5B and Fig. S8A). Both GRP expression and ASCL1 expression were strongly reduced in Cluster 2 after treatment (Fig. S7B). In addition, the mean expression of neuroendocrine markers (NE score) including ASCL1, CHGA, GRP, INSM1, NCAM1, FOXA2, and SOX2 was significantly reduced in Cluster 2 after treatment (Fig. 5C, left), suggesting cells in this cluster are losing NE features. On the other hand, the mean expression of 76 EMT marker genes from the Broad MSigDB Hallmark Signatures (EMT score) was upregulated in multiple cell clusters after treatment, but most significantly increased in Cluster 0 (Fig. 5C, right). This observation is supported by the significant upregulation of EMT and lung cancer survival pathways in Cluster 0 after treatment (Fig. S8B). Altogether, these results highlight the loss of NE cell subpopulation and the emergence of several transcriptionally distinct, mesenchymal‐like populations in NCI‐H69 cells resistant to LSD1 inhibitors.

In an attempt to assess the reversibility of the mesenchymal shift observed in LSD1 inhibitor persister cells, we performed drug washout experiments. After 14 days of GSK690 treatment in NCI‐H526 and NCI‐H69 cells, antiproliferative effects occurred concomitant with the upregulation of mesenchymal markers CDH2, SNA1, MYC, VIM, and ZEB1 and the downregulation of neuroendocrine markers GRP, ASCL1, and CHGA (Fig. S9). After 7 days of drug washout, gene expression signatures recovered in both cell lines to levels similar to that observed in DMSO controls. Moreover, after 7 days of drug washout, cells regained sensitivity to LSD1 inhibition to a similar extent as untreated cell lines (Fig. S9). These data suggest that the mesenchymal shift in SCLC cells treated with LSD1 inhibitors is reversible and caused by an ‘epi‐stable’ mesenchymal‐like differentiation state.

### 3.6 LSD1 inhibitor resistance occurs through chromatin accessibility changes for genes associated with neuroendocrine‐ and mesenchymal‐like programs

**Fig. 6:**
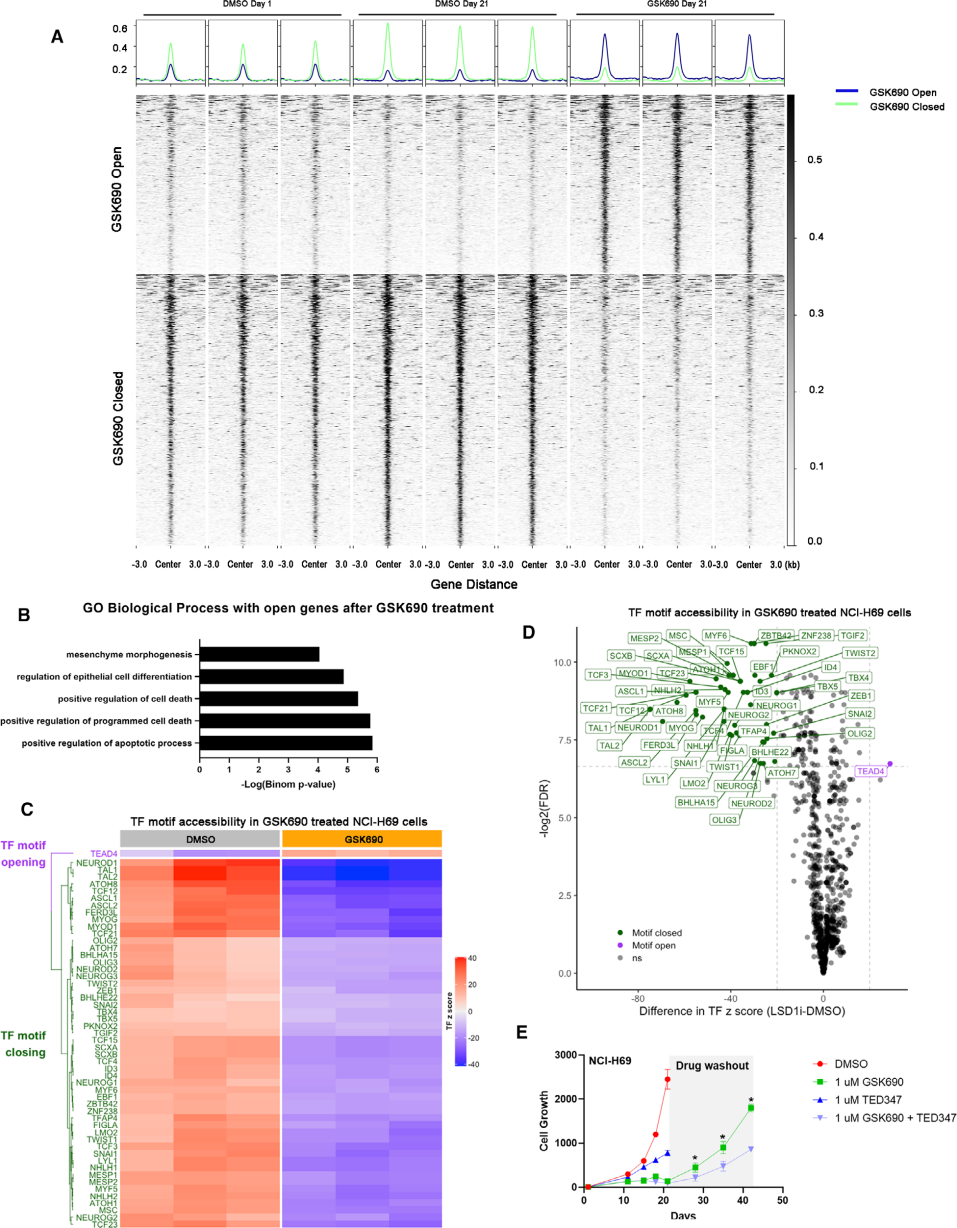
Chromatin accessibility changes for genes associated with neuroendocrine and mesenchymal expression signatures (A) ATAC‐seq signal at differentially open or closed regions (FDR < 1% and fold change > 2.5) between vehicle (DMSO day 0 and day 21) or 0.3 μm GSK690 (day 21) in NCI‐H69 cells (*n* = 3 biologically independent replicates). (B) GO Biological Process using GREAT analysis with open genes associated with 0.3 μm GSK690 treatment. (C) Heatmap representing TF *z*‐scores of significantly open or closed TF DNA binding motifs (*n* = 3 biologically independent replicates). (D) Volcano plot showing the significantly open or closed TF DNA binding motifs (FDR < 1% and difference in mean *z*‐score > 20) with 0.3 μm GSK690 using chromVar analysis. (E) Growth curves of NCI‐H69 cells treated with either 1 µm LSD1 inhibitor GSK690, or 1 µm TED‐347, or combination of 1 µm GSK690 with 1 µm TED‐347 for 21 days followed by 21‐day drug washout (mean ± SD with *n* = 3 biologically independent replicates). Two‐way ANOVA multiple comparisons with *P‐values ≤ 0.001 between 1 µm GSK690 and 1 µm GSK690 plus 1 µm TED‐347 are shown.

We next sought to define the extent of epigenetic reprogramming in LSD1 inhibitor drug‐tolerant cells by employing an assay for transposase‐accessible chromatin using sequencing (ATAC‐seq). ATAC‐seq data in NCI‐H69 cells treated with 0.3 μm GSK690 for 21 days were highly reproducible between biological replicates and showed clear enrichment at specific genomic regions (Fig. S10A). Consistent with observed changes in gene expression, differentially open or closed chromatin accessibility peaks were primarily identified at distal regulatory regions of genes after 21 days of GSK690 treatment compared with controls (Fig. 6A, Fig. S10B). GREAT gene ontology analysis [39] for genes acquiring open chromatin accessibility peaks near regulatory regions suggests enrichment in pathways involved in mesenchyme morphogenesis and regulation of epithelial cell differentiation (Fig. 6B). In contrast, genes that acquire closed chromatin do not show any significant pathway enrichment.

To study the transcription factor programs regulating drug‐resistant SCLC cells, we identified the transcription factor motifs represented in differentially accessible, closed or open, chromatin regions after GSK690 treatment (Fig. 6C,D). We used chromvar [40], a package designed for inferring transcription factor activity from ATAC‐seq based on chromatin accessibility levels at TF binding motifs genome‐wide (Fig. 6C,D). Our analysis demonstrated that NEUROD1/NEUROD2 and ASCL1 DNA binding motifs were significantly closed following GSK690 treatment. The DNA binding motif for TEAD4 is the only significantly opened region with the treatment (Fig. 6C,D). TEAD4 is a downstream mediator of YAP1 activity, which has been previously identified as a possible phenotypic modulator in a subset of non‐neuroendocrine SCLC cell lines [48]. A small molecule, TED‐347, was recently discovered to demonstrate covalent engagement of cysteine to inhibit TEAD4·YAP1 protein–protein interaction and block TEAD transcriptional activity [49]. We treated NCI‐H69 cells with either LSD1 inhibitor GSK690 or combination with TED‐347 for 21 days. Combination treatment with TED‐347 significantly prevented the cell regrowth following the drug withdrawal for up to 21 days (Fig. 6E). Thus, inhibiting TEAD transcriptional activity prevents the acquired resistance of SCLC in response to LSD1 inhibitors. These data indicate that mesenchymal‐like drug tolerance in SCLC cell lines occurs through epigenetic reprogramming mediated through changes in transcription factor programs driven by oncogenic YAP signaling.

## 4 Discussion

In this study, we have identified common features of intrinsic and acquired drug resistance to LSD1 inhibitors through epigenetic changes in SCLC neuroendocrine transcriptional programs. Epigenetic plasticity is known to contribute to the poor durability of responses of SCLC to current therapies as selection of mesenchymal enriched clones become enriched in refractory disease found in relapse [50, 51]. Many SCLC cell lines analyzed in this study show either no response or only partial response to LSD1 inhibitors even after 21 days of treatment, suggesting many SCLC cell lines either have a preexisting drug‐resistant population or are capable of transitioning to a drug‐resistant state. Experimentally neuroendocrine SCLC cell lines can transition from neuroendocrine into mesenchymal phenotypes when treated with targeted or chemotherapies [19, 44, 45, 50]. This observation is clinically relevant as SCLC tumors are often composed of epigenetically heterogeneous cells with either a neuroendocrine or a mesenchymal profile [18, 19, 50].

Despite early literature suggesting LSD1 inhibitors would be broadly active in numerous cancers including colon, prostate, and breast cancer [12, 52, 53], the generation of more selective LSD1 inhibitors has clarified the extent of LSD1 inhibitor activity primarily in AML and SCLC [5, 54]. One possible explanation for this discrepancy is suggested from the recent reports identifying noncatalytic mechanisms of action of current LSD1 inhibitors being explored in the clinic. Inhibition of the interaction of LSD1 with SNAG domain‐containing proteins INSM1 or GFI1B impacts expression of key neuroendocrine lineage transcriptional regulators in SCLC including ASCL1 [15]. Recently, a specific inhibitor of LSD1 enzyme activity has been discovered, T‐448 [55], which has minimal impact on the interaction of the LSD1‐CoRest complex with SNAG domain transcription factors. In our studies, T‐448 has no antiproliferative effect on NCI‐H69 cells after 21‐day treatment (Fig. S11), suggesting that the mechanism of action of LSD inhibitors occurs primarily through disruption of the interaction of LSD1 with SNAG domain transcription factors. Our data demonstrate that all cell lines sensitive to LSD1 inhibitors express either INSM1 or GFI1B; however, a subset of cell lines resistant to the drug also express these genes. Thus, the expression of these SNAG domain‐containing proteins alone does not predict sensitivity to LSD1 inhibitors. Interestingly, LSD1 has been found to interact with the SNAG domain of SNAIL and implicated in the transcriptional repression of epithelial markers during EMT transition in breast cancer cells [56]. The role of LSD1 in the SNAG domain of targeted proteins appears to be context and lineage specific.

Our results demonstrate that SCLC cells which have evolved non‐neuroendocrine, mesenchymal‐like transcriptional programs are resistant to LSD1 inhibition. These findings are consistent with Mohammad *et al*., [5] who identified TGF‐beta pathway signatures negatively correlate with LSD1 inhibitor sensitivity in SCLC. The TGF‐beta pathway is known to regulate EMT pathways in cancer as well as in normal development [46]. In cell lines sensitive to LSD1 inhibitors, we did observe enrichment for TGF‐beta pathway genes as well as genes involved in axonal and EMT pathways after LSD1 inhibitor treatment. Therefore, these data are potentially consistent with our observation that neuroendocrine and mesenchymal differentiation markers stratify sensitivity of SCLC cell lines to LSD1 inhibitors. Augert *et al*. also identified heterogeneous responses to the LSD1 inhibitor ORY‐1001 in a screen of SCLC patient‐derived xenograft (PDX) models [13]. In their study, PDX models capable of NOTCH activation and concomitant ASCL1 downregulation were enriched in response to LSD1 inhibition. In gene expression studies following GSK690 treatment, we did observe induction of NOTCH pathway genes in COR‐L88 and NCI‐H1417 cells but not to the same extent in NCI‐H69 or NCI‐H889 cells. This suggests that NOTCH pathway‐independent mechanisms of LSD1 inhibitor response exist in SCLC.

This study highlights the remarkable plasticity of SCLC to evolve into drug‐resistant mesenchymal‐like states. Along these lines, we found expression of the neuroendocrine transcription factor ASCL1 was downregulated after GSK690 treatment in most models with the exception of NCI‐H526 cells which shows a very low expression level of ASCL1 and is reported that the interaction between LSD1 and SNAG domain protein GFI1B plays a key role in this cell line [15]. The extent of mesenchymal gene induction in GSK690‐treated SCLC cells does appear comparatively lower relative to what we observed in mesenchymal‐shifted NCI‐H69V cells and thus may represent a ‘partial EMT’ [47]. In support of this model, drug washout experiments suggest these changes are ‘epi‐stable’ as cells revert to neuroendocrine phenotypes within 7 days of drug removal. Additionally, we found many SCLC cell lines analyzed in this study show only partial response to LSD1 inhibitors even after 21 days of treatment, suggesting many SCLC cell lines are capable of transitioning to a drug‐resistant state. Single cell RNA‐seq suggests that this resistant population likely represents an acquired drug‐tolerant state represented by transcriptional reprogramming that is maintained under drug selection. Consistent with this finding, ATAC‐seq data in NCI‐H69 cells highlighted a shift in the utilization of transcription factors from ASCL1 to TEAD4 in drug‐resistant cells. Interestingly, the expression of TEAD4 and downstream YAP and TAZ have recently been highlighted to represent a unique subtype of non‐ASCL1‐driven SCLC [48]. Our data suggest that inhibiting TEAD transcriptional activity may be an effective strategy to prevent the acquired resistance of SCLC in response to LSD1 inhibitors.

A recent phase I study of GSK2879552 in relapsed or refractory SCLC found a disease control rate of 14% (four of 29 patients) [57]. High rates of adverse events, including thrombocytopenia and encephalopathy, lead to the termination of the study. Our data suggest that only a segment of SCLC patients, with a defined neuroendocrine differentiation state, will likely benefit from LSD1 inhibitors. In LSD1 inhibitor‐treated patients, a mesenchymal‐like resistant population is likely to persist. Thus, emphasis should focus on drug combination approaches that can sustain clinical responses by eliminating cells that maintain residual disease. Experimentally, it has been demonstrated that SCLC cells treated with chemotherapy [50] or radiotherapy [58] obtain mesenchymal characteristics that confer drug resistance. Thus, the efficacy of LSD1 inhibitors in SCLC patients heavily pretreated with chemotherapy will be an important consideration. The recent disclosures of additional LSD1 inhibitors, IMG‐7289, INCB059872, and CC‐90011, under clinical development for SCLC highlight the importance of clarifying the mechanism of action as well as further defining the clinical application of these drugs [6].

## 5 Conclusions

We discovered that epigenetic plasticity contributes to heterogeneous responsiveness of SCLC to LSD1 inhibitors. Sensitivity to LSD1 inhibitors in SCLC is confined primarily to cells that express neuroendocrine transcriptional programs. Selection of a TEAD4‐driven mesenchymal‐like subpopulation is likely to present a barrier to effective single‐agent responses in the clinic. Drug combinations targeting the YAP‐TEAD pathways in combination with LSD1 inhibitors may be an effective strategy to target intrinsic and adaptive mesenchymal‐like resistant populations and sustain effective clinical responses.

## Conflict of interest

WY, C‐YC, SS, TX, MO, TN, JF, and TAP are employed by and hold stock in Pfizer, Inc. No potential conflicts of interests were disclosed by the other authors.

## Author contributions

WY designed and executed experiments; collected, analyzed, and interpreted data and contributed to writing and editing the manuscript. C‐YC performed bioinformatic analyses; analyzed and interpreted data; and contributed to writing the manuscript. TX performed bioinformatic analyses. MO executed experiments and analyzed data. TCN and JF executed and analyzed histopathology. ARU reviewed and provided input on the contents of the manuscript. SS designed the study, analyzed, and interpreted data and contributed to writing the manuscript. TAP designed the study and wrote the manuscript. All authors reviewed and approved the final manuscript.

### Peer Review

The peer review history for this article is available at https://publons.com/publon/10.1002/1878‐0261.13124.

## Supporting information


**Fig. S1.** Growth rates of NCI‐H526 (A, left) and DMS114 (B, left) cells stably expressing either control shRNA or two independent shRNAs targeting LSD1 following 4, 7, or 14 days post‐infection with lentiviral vector. Knockdown of LSD1 was assessed by western blot at day 4 post‐infection in NCI‐H526 (A, right) and DMS114 (B, right) cells. Two‐way ANOVA multiple comparisons with p‐values are shown. Lentiviral shRNA vectors pRSI12‐U6‐(sh)‐UbiC‐TagRFP‐2A‐Puro were purchased from Cellecta with targeting sequences for non‐targeting (CAACAAGATGAAGAGCACCAA), LSD1 shRNA #1 (CCAACAATTAGAAGCACCTTA) and LSD1 shRNA #2 (AGGAAGGCTCTTCTAGCAATA). Each shRNA lentiviral expression construct was packaged with lentiviral packaging mix (Thermo Scientific) in 293T cells according to manufacturer’s instructions. Cell growth was monitored by CellTiter‐Glo Luminescent Cell Viability Assay (Promega) at day 7 and day 14 from experimental triplicates.
**Fig. S2.** Structures and profile of GSK690 (R)‐4‐(5‐(pyrrolidin‐3‐ylmethoxy)‐2‐(p‐tolyl)pyridin‐3‐yl)benzonitrile and OG‐86 (1S,2R)‐N‐((2‐methoxypyridin‐3‐yl)methyl)‐2‐phenylcyclopropan‐1‐amine compounds based off publicly available data.
**Fig. S3.** Long‐term proliferation assays for NCI‐H1417 (A), NCI‐H187 (B), NCI‐H889 (C), and DMS‐114 (D) cells treated with vehicle (DMSO), 0.3 µM or 1.0 µM GSK690, or 0.3 µM or 1.0 µM OG‐86. (E) Cell cycle analysis of NCI‐H526, COR‐L88, and NCI‐H1417 cells after 7 days of treatment with DMSO or indicated concentrations of GSK690. (F) Cell cycle analysis by propidium iodide staining in COR‐L88 cells treated with DMSO, 0.3 µM, or 1 µM GSK690 for 14 days. Cell number was calculated from experimental triplicates by cell counting at indicated time points. Two‐way ANOVA multiple comparisons with p‐values are shown.
**Fig. S4.** Gene expression (Log2) and copy number of MYC, MYCN, and MYCL in GSK690 sensitive and resistant SCLC cell lines.
**Fig. S5.** (A) Principal component analysis of cell line gene expression derived from CCLE cell line data showing segregation of GSK690 sensitive models (green) and resistant models (red) on PC1 vs. PC2. (B) Venn diagram showing overlap of DE gene signature from Figure 3A with previously published NE or ML signature genes [1]. (C) Pathway analysis of genes altered in expression (FDR < 0.05, fold change ≥ 2) by GSK690 treatment utilizing MSigDB pathway databases. Top 10 enriched pathways are shown with the p‐value cutoff of 0.05.
**Fig. S6.** Western Blot analysis of neuroendocrine and mesenchymal protein levels using indicated antibodies in SCLC cells lines treated with DMSO or 0.3 µM GSK690 for 14 days.
**Fig. S7.** (A) Left: cluster number selection within UMAP using resolution = 0.15. To optimize single cell clustering, we tuned the “resolution” parameter in Seurat, and selected resolution = 0.15 by visual inspection and the presence of robust differential markers between clusters. Right: cell number change in each cluster depicted as percentage of total cells in each cluster before and after GSK690 treatment. (B) Expression of ASCL1 or GRP in cluster 2 in UMAP or as violin plots following treatment with vehicle (DMSO) or 0.3 μM GSK690 for 21 days.
**Fig. S8.** (A) Top 10 uniquely expressed genes in each single cell RNA‐seq cluster. The cluster specific genes were identified by performing DE analyses between each cluster with all the rest of clusters. DE cutoff is FDR < 5% and logFC > 0.25. (B) For differential pathway analysis, a hypergeometric test with FDR correction was applied to cluster specific differential genes using the MSigDB data base. NE (8 genes) or EMT (76 genes) gene score were calculated as the mean expression value of the detected gene sets in each single cell, and two sample t‐test was performed to calculate the significance between DMSO and treatment. Pathways analysis showed top 10 up‐regulated pathways within each cluster following treatment of 0.3 μM GSK690 for 21 days. P‐value cutoff = 0.05. Cluster 3 and 4 did not have any significant enrichment.
**Fig. S9.** Cell viability and gene expression of neuroendocrine and mesenchymal markers in NCI‐H526 and NCI‐H69 cells pre‐treated with 0.3 µM GSK690 for 14 days and following 7‐day washout.
**Fig. S10.** (A) Representative ATAC‐seq profiles of biological replicates from day 0, day 21 DMSO or 0.3 µM GSK690 treatment. (B) Percentage of ATAC‐seq peaks in cells treated with GSK690, separated into promoter (<±3 kb transcription start site [TSS]) and distal regions (>±3 kb TSS).
**Fig. S11.** Long‐term proliferation assay for NCI‐H69 cells treated with T‐448 with a dose‐range up to 1.8 uM for 21 days.

## Data Availability

All data generated and/or analyzed during this study are available within this manuscript or as Supplementary Information and from the corresponding authors.
