# Supplementary material for: Intrinsic and acquired drug resistance to LSD1 inhibitors in small cell lung cancer occurs through a TEAD4‐driven transcriptional state
Source: Mol Oncol. 2021 Nov 9;16(6):1309–28. doi: 10.1002/1878-0261.13124 (PMC8936524; doi:10.1002/1878-0261.13124)
Supplement: Supplementary file 1 — Fig. S1. Growth rates of NCI‐H526 (A, left) and DMS114 (B, left) cells stably expressing either control shRNA or two independent shRNAs targeting LSD1 following 4, 7, or 14 days post‐infection with lentiviral vector. Knockdown of LSD1 was assessed by western blot at day 4 post‐infection in NCI‐H526 (A, right) and DMS114 (B, right) cells. Two‐way ANOVA multiple comparisons with p‐values are shown. Lentiviral shRNA vectors pRSI12‐U6‐(sh)‐UbiC‐TagRFP‐2A‐Puro were purchased from Cellecta with targeting sequences for non‐targeting (CAACAAGATGAAGAGCACCAA), LSD1 shRNA #1 (CCAACAATTAGAAGCACCTTA) and LSD1 shRNA #2 (AGGAAGGCTCTTCTAGCAATA). Each shRNA lentiviral expression construct was packaged with lentiviral packaging mix (Thermo Scientific) in 293T cells according to manufacturer’s instructions. Cell growth was monitored by CellTiter‐Glo Luminescent Cell Viability Assay (Promega) at day 7 and day 14 from experimental triplicates. Fig. S2. Structures and profile of GSK690 (R)‐4‐(5‐(pyrrolidin‐3‐ylmethoxy)‐2‐(p‐tolyl)pyridin‐3‐yl)benzonitrile and OG‐86 (1S,2R)‐N‐((2‐methoxypyridin‐3‐yl)methyl)‐2‐phenylcyclopropan‐1‐amine compounds based off publicly available data. Fig. S3. Long‐term proliferation assays for NCI‐H1417 (A), NCI‐H187 (B), NCI‐H889 (C), and DMS‐114 (D) cells treated with vehicle (DMSO), 0.3 µM or 1.0 µM GSK690, or 0.3 µM or 1.0 µM OG‐86. (E) Cell cycle analysis of NCI‐H526, COR‐L88, and NCI‐H1417 cells after 7 days of treatment with DMSO or indicated concentrations of GSK690. (F) Cell cycle analysis by propidium iodide staining in COR‐L88 cells treated with DMSO, 0.3 µM, or 1 µM GSK690 for 14 days. Cell number was calculated from experimental triplicates by cell counting at indicated time points. Two‐way ANOVA multiple comparisons with p‐values are shown. Fig. S4. Gene expression (Log2) and copy number of MYC, MYCN, and MYCL in GSK690 sensitive and resistant SCLC cell lines. Fig. S5. (A) Principal component analysis of cell line gene expression derived from CCL [file MOL2-16-1309-s001.docx]

**Supplemental Figures Legends**

Figure S1: Growth rates of NCI-H526 (A, left) and DMS114 (B, left) cells stably expressing either control shRNA or two independent shRNAs targeting LSD1 following 4, 7, or 14 days post-infection with lentiviral vector. Knockdown of LSD1 was assessed by western blot at day 4 post-infection in NCI-H526 (A, right) and DMS114 (B, right) cells. Two-way ANOVA multiple comparisons with p-values are shown. Lentiviral shRNA vectors pRSI12-U6-(sh)-UbiC-TagRFP-2A-Puro were purchased from Cellecta with targeting sequences for non-targeting (CAACAAGATGAAGAGCACCAA), LSD1 shRNA #1 (CCAACAATTAGAAGCACCTTA) and LSD1 shRNA #2 (AGGAAGGCTCTTCTAGCAATA). Each shRNA lentiviral expression construct was packaged with lentiviral packaging mix (Thermo Scientific) in 293T cells according to manufacturer’s instructions. Cell growth was monitored by CellTiter-Glo Luminescent Cell Viability Assay (Promega) at day 7 and day 14 from experimental triplicates.

Figure S2: Structures and profile of GSK690 (R)-4-(5-(pyrrolidin-3-ylmethoxy)-2-(p-tolyl)pyridin-3-yl)benzonitrile and OG-86 (1S,2R)-N-((2-methoxypyridin-3-yl)methyl)-2-phenylcyclopropan-1-amine compounds based off publicly available data.

Figure S3: Long-term proliferation assays for NCI-H1417 (A), NCI-H187 (B), NCI-H889 (C), and DMS-114 (D) cells treated with vehicle (DMSO), 0.3 μM or 1.0 μM GSK690, or 0.3 μM or 1.0 μM OG-86. (E) Cell cycle analysis of NCI-H526, COR-L88, and NCI-H1417 cells after 7 days of treatment with DMSO or indicated concentrations of GSK690. (F) Cell cycle analysis by propidium iodide staining in COR-L88 cells treated with DMSO, 0.3 μM, or 1 μM GSK690 for 14 days. Cell number was calculated from experimental triplicates by cell counting at indicated time points. Two-way ANOVA multiple comparisons with p-values are shown.

Figure S4: Gene expression (Log2) and copy number of MYC, MYCN, and MYCL in GSK690 sensitive and resistant SCLC cell lines. Expression and copy number were determined from CCLE data for cell lines used in analysis. Significance was calculated using an unpaired t-test and samples with p<0.05 are indicated.

Figure S5: (A) Principal component analysis of cell line gene expression derived from CCLE cell line data showing segregation of GSK690 sensitive models (green) and resistant models (red) on PC1 vs. PC2. (B) Venn diagram showing overlap of DE gene signature from Figure 3A with previously published NE or ML signature genes [[1](#_ENREF_1)]. (C) Pathway analysis of genes altered in expression (FDR < 0.05, fold change >=2) by GSK690 treatment utilizing MSigDB pathway databases. Top 10 enriched pathways are shown with the p-value cutoff of 0.05.

Figure S6: Western Blot analysis of neuroendocrine and mesenchymal protein levels using indicated antibodies in SCLC cells lines treated with DMSO or 0.3 μM GSK690 for 14 days.

Figure S7: (A) Left: cluster number selection within UMAP using resolution = 0.15. To optimize single cell clustering, we tuned the “resolution” parameter in Seurat, and selected resolution = 0.15 by visual inspection and the presence of robust differential markers between clusters. Right: cell number change in each cluster depicted as percentage of total cells in each cluster before and after GSK690 treatment. (B) Expression of ASCL1 or GRP in cluster 2 in UMAP or as violin plots following treatment with vehicle (DMSO) or 0.3 μM GSK690 for 21 days.

Figure S8: (A) Top 10 uniquely expressed genes in each single cell RNA-seq cluster. The cluster specific genes were identified by performing DE analyses between each cluster with all the rest of clusters. DE cutoff is FDR < 5% and logFC > 0.25. (B) For differential pathway analysis, a hypergeometric test with FDR correction was applied to cluster specific differential genes using the MSigDB data base. NE (8 genes) or EMT (76 genes) gene score were calculated as the mean expression value of the detected gene sets in each single cell, and two sample t-test was performed to calculate the significance between DMSO and treatment. Pathways analysis showed top 10 up-regulated pathways within each cluster following treatment of 0.3 μM GSK690 for 21 days. P-value cutoff = 0.05. Cluster 3 and 4 did not have any significant enrichment.

Figure S9: Cell viability and gene expression of neuroendocrine and mesenchymal markers in NCI-H526 and NCI-H69 cells pre-treated with 0.3 μM GSK690 for 14 days and following 7-day washout. NCI-H526 and NCI-H69 cells were treated in triplicate for 14 days with DMSO or 0.3 µM GSK690. Every 3 or 4 days, adherent and suspension cells were collected and 2 ml of cells was transferred to 10cm2 dishes for subsequent culture. 8 ml of fresh cell culture medium was added to each plate and drug was added at proper concentrations to each 10 cm dish. After removal of drug for 7 days, remaining cells from the previously pre-treated population with DMSO or 0.3 µM GSK690 were plated at similar concentrations in triplicate. Cells were then exposed to DMSO, 0.1 µM, 0.3 µM, or 1.0 µM of GSK690 and counted at 7, 10, and 14 days following re-exposure to drug. At each time-point, three aliquots of 1 ml of cells was collected in 1.5 ml microcentrifuge tube, concentrated by centrifugation to a 100 µl volume and added to a 96 well plate. Relative cell numbers were determined by cell titer-glo reagent (Promega). Aliquots were also removed at several indicated time points for RNA purification using RNeasy kit (Qiagen). Gene expression of SYP, GRP, NCAM, CHGA, OVOL2, ASCL1, CHD1, CHD2, SNA1, ZEB1, VIM, and MYC were assessed by qRT-PCR using indicated taqman gene expression assays (Applied Biosystems). Expression of each sample was normalized to GAPDH and delta-Ct values calculated from fold change relative to DMSO at each time point. Two-way ANOVA multiple comparisons with p-values are shown.

Figure S10: (A) Representative ATAC-seq profiles of biological replicates from day 0, day 21 DMSO or 0.3 μM GSK690 treatment. (B) Percentage of ATAC-seq peaks in cells treated with GSK690, separated into promoter (<±3 kb transcription start site [TSS]) and distal regions (>±3 kb TSS).

Figure S11: Long-term proliferation assay for NCI-H69 cells treated with T-448 with a dose-range up to 1.8 uM for 21 days. Cell growth was monitored by CellTiter-Glo Luminescent Cell Viability Assay (Promega) at day 21 from experimental triplicates.

**Supplemental Figures**

Figure S1


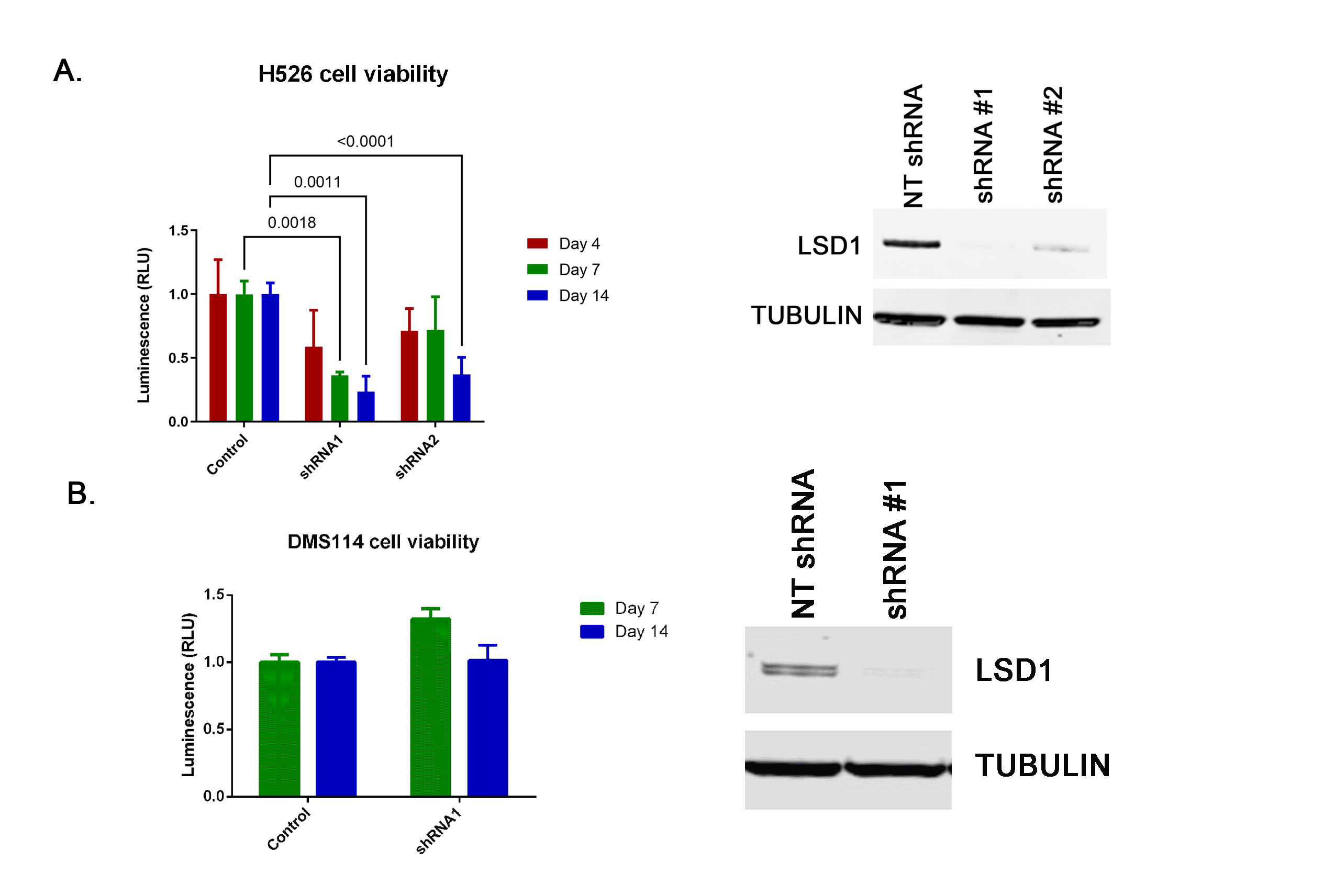


Figure S2


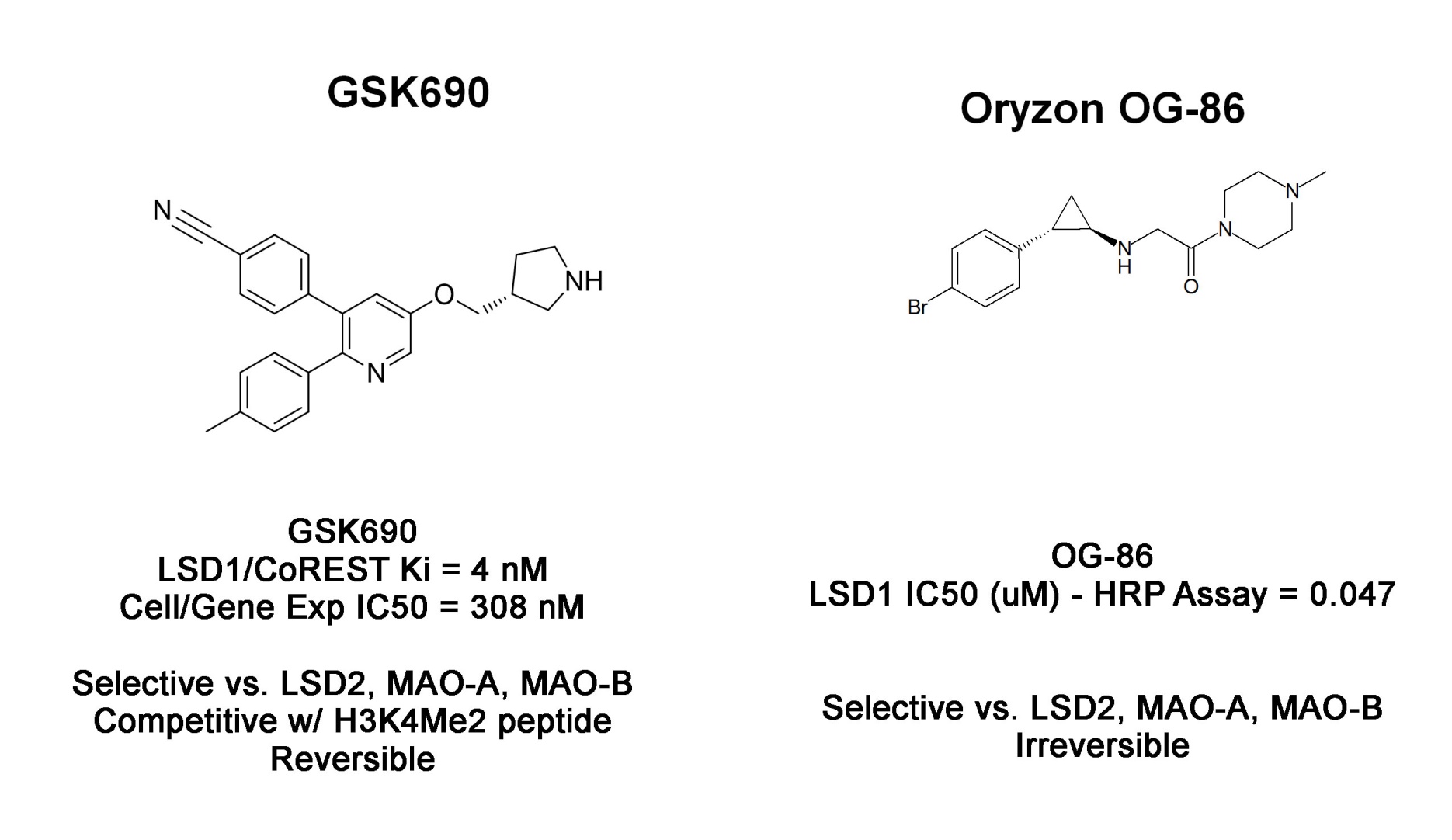


Figure S3


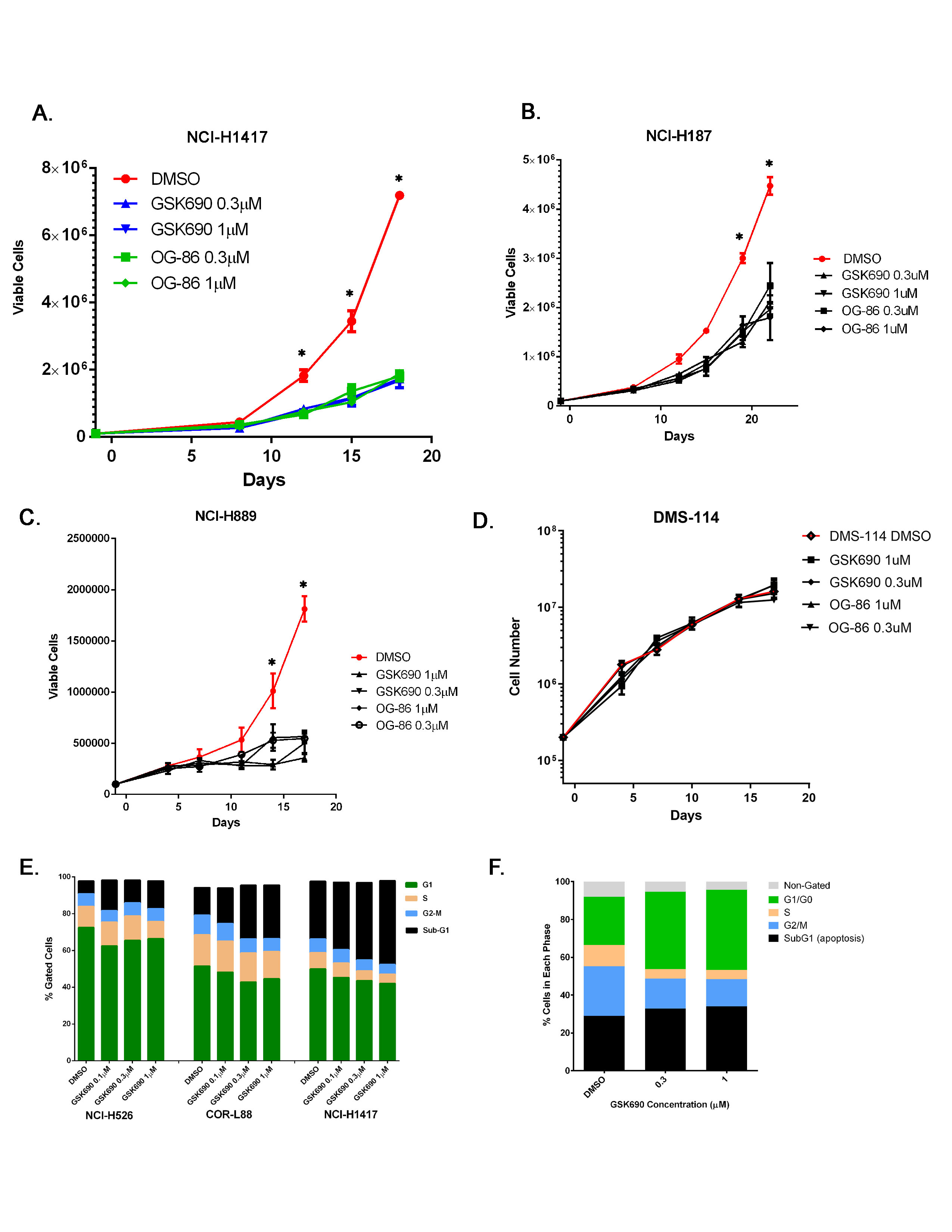


Figure S4


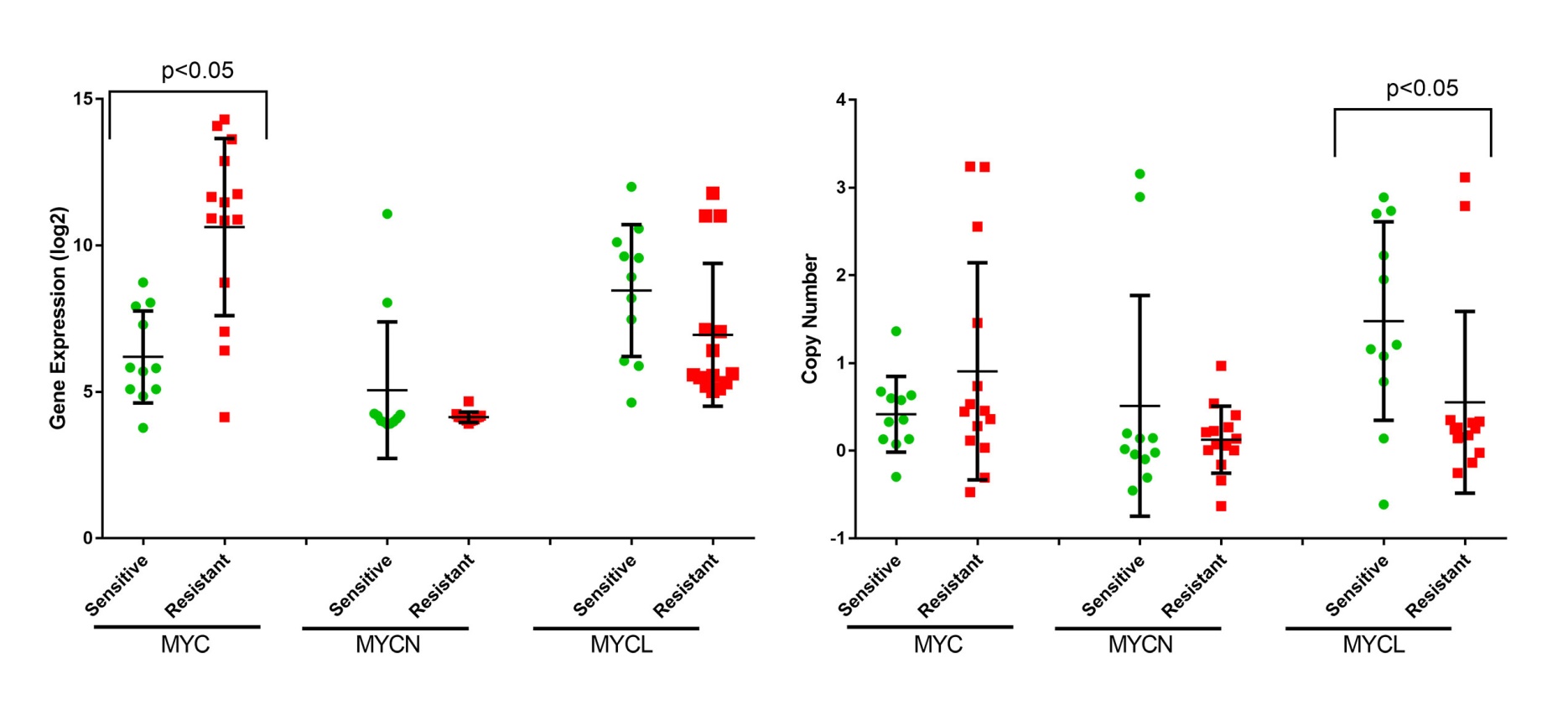


Figure S5


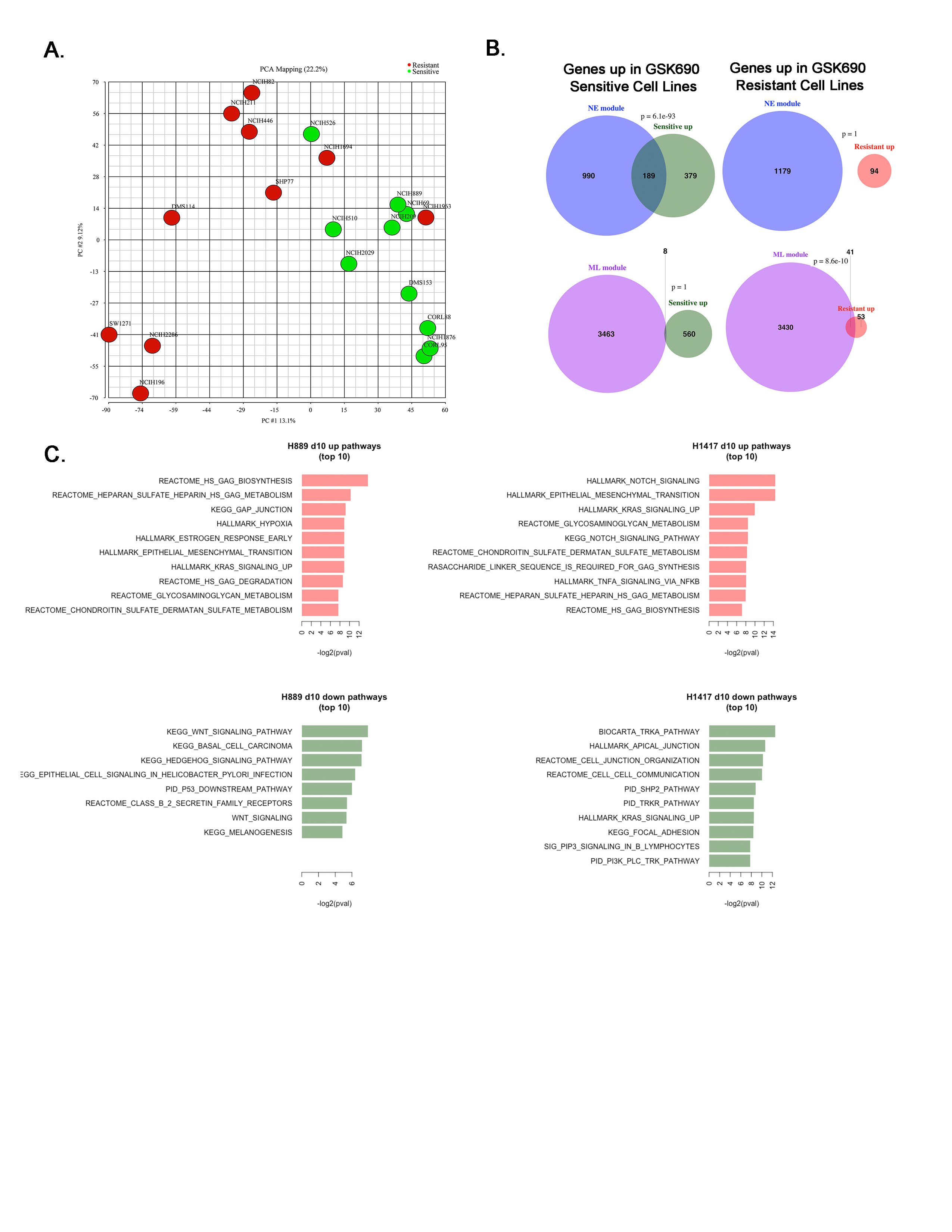


Figure S6


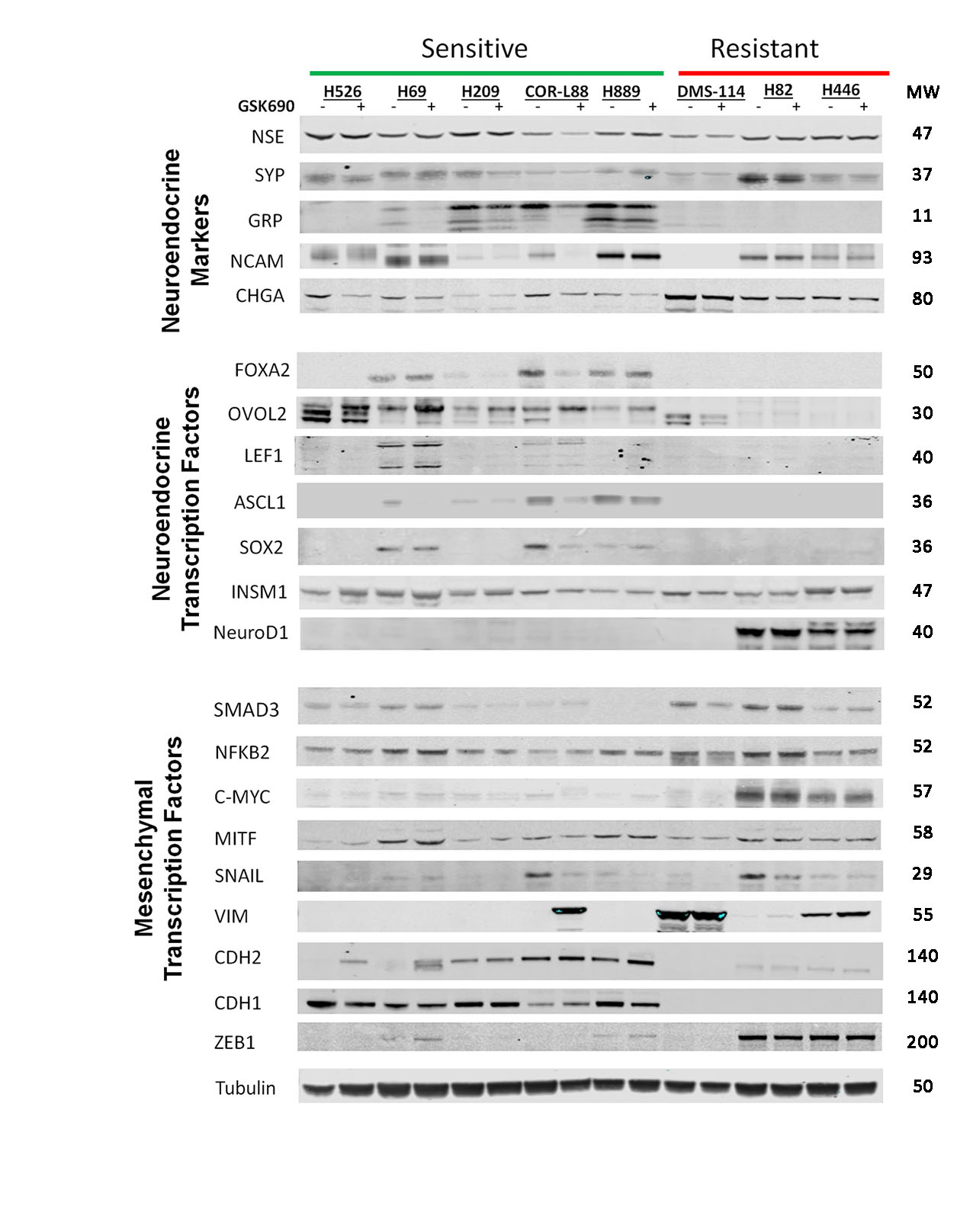


Figure S7


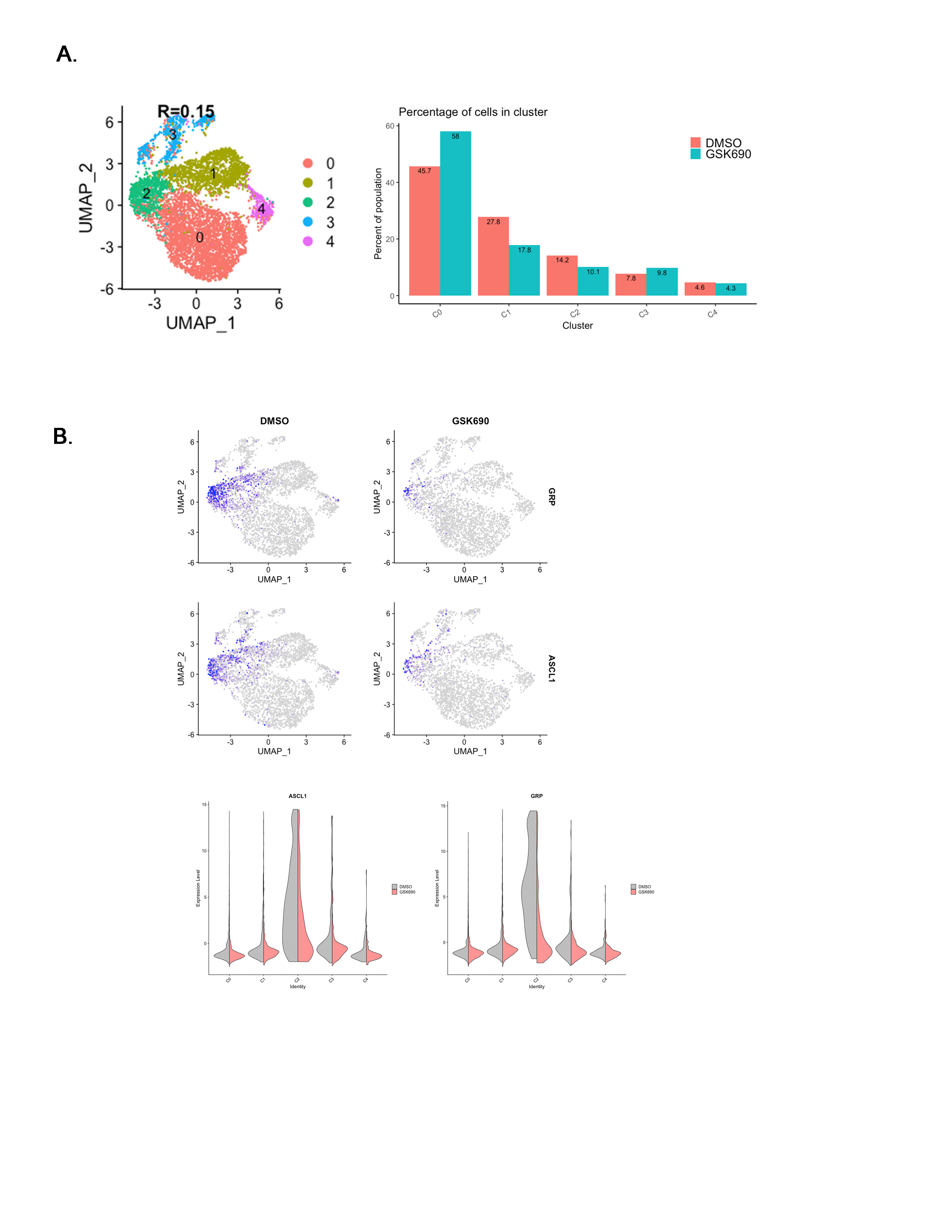


Figure S8

Figure S9


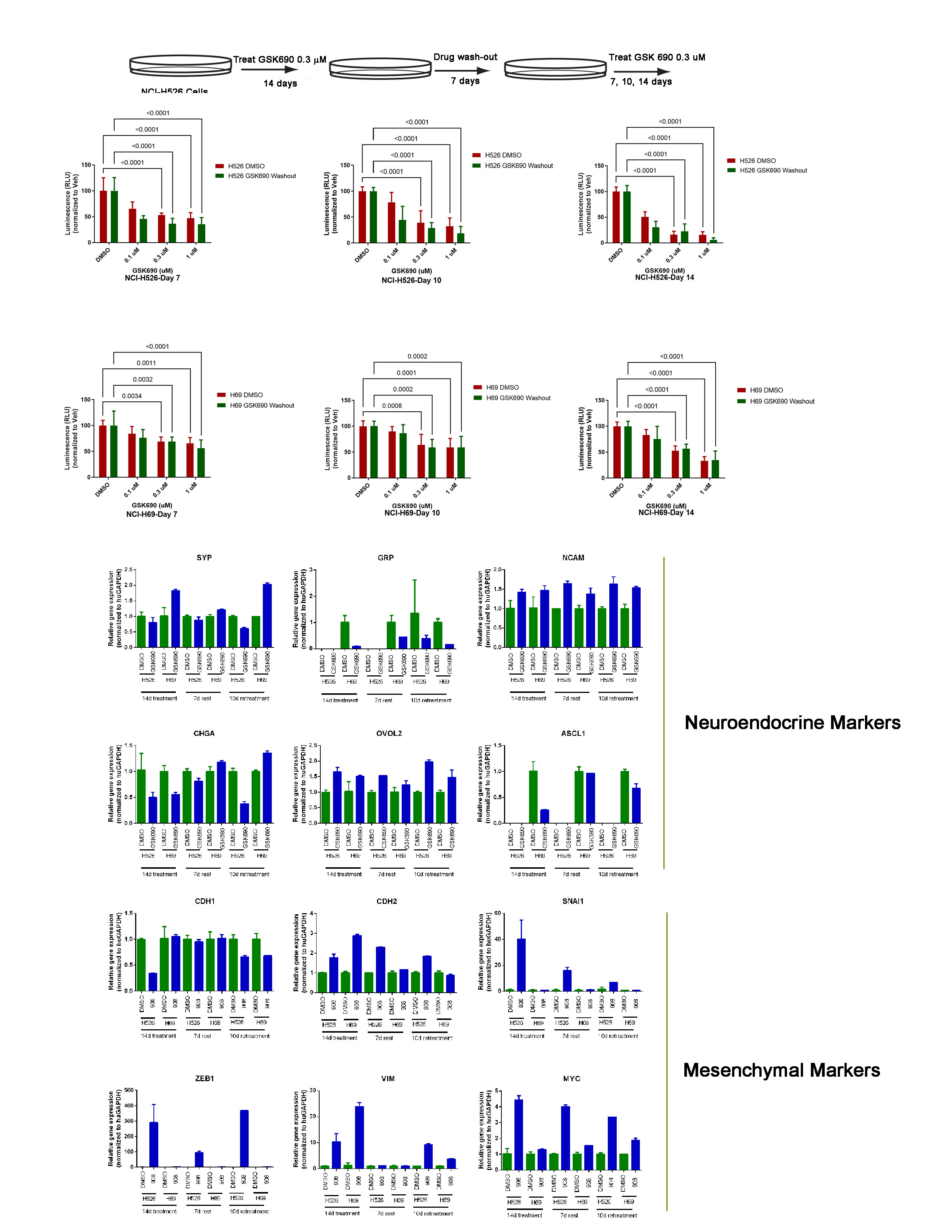


Figure S10

A.
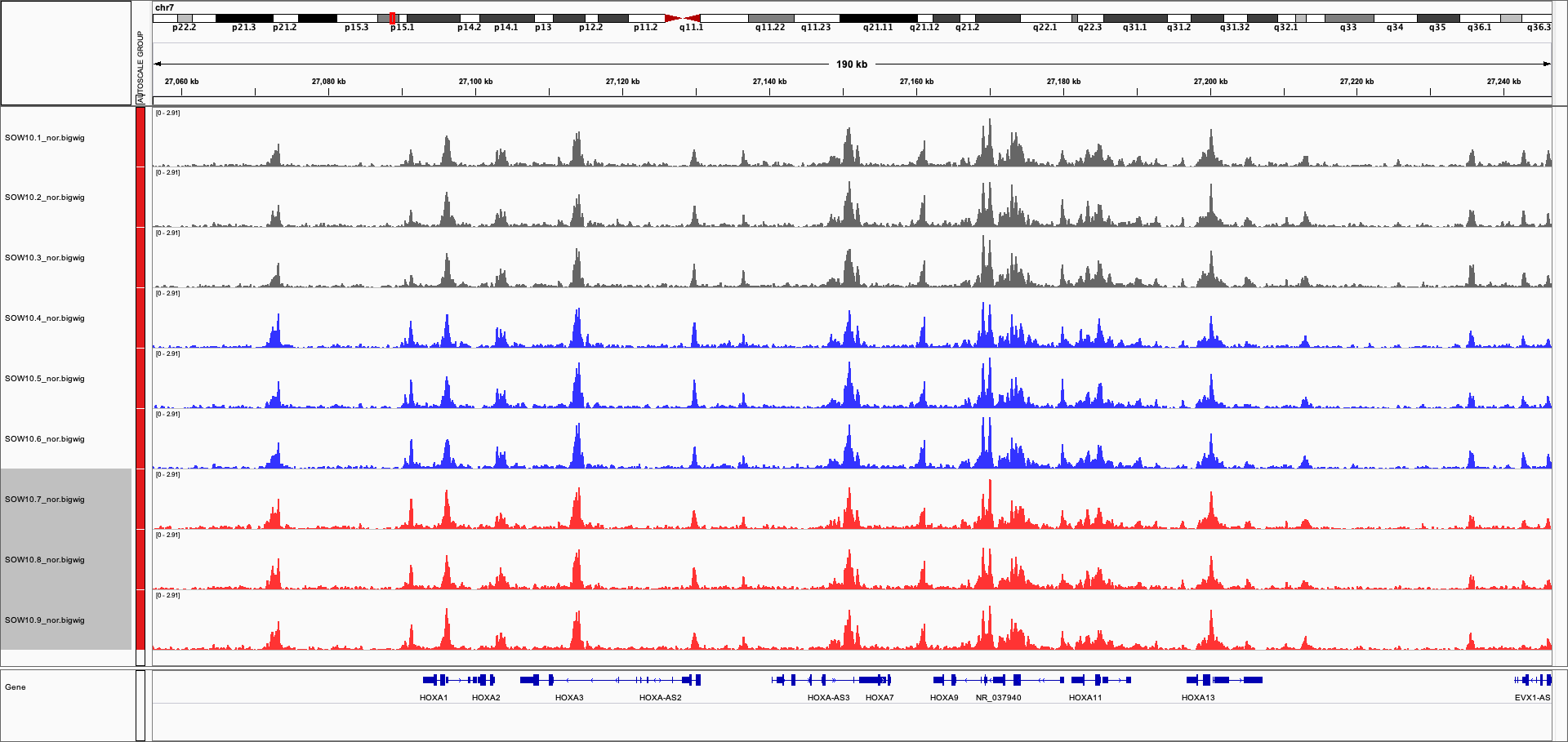

Figure S11
